# Heterogeneity of Synchronous Lung Metastasis Calls for Risk Stratification and Prognostic Classification: Evidence from a Population-Based Database

**DOI:** 10.3390/cancers14071608

**Published:** 2022-03-22

**Authors:** Shuncong Wang, Lei Chen, Yuanbo Feng, Johan V. Swinnen, Charles Jonscher, Chantal Van Ongeval, Yicheng Ni

**Affiliations:** 1 Theragnostic Laboratory, KU Leuven, Biomedical Group, Campus Gasthuisberg, 3000 Leuven, Belgium; shuncong.wang@kuleuven.be (S.W.); lei.chen@kuleuven.be (L.C.); yuanbo.feng@kuleuven.be (Y.F.); 2 Laboratory of Lipid Metabolism and Cancer, KU Leuven, Biomedical Group, Campus Gasthuisberg, 3000 Leuven, Belgium; j.swinnen@kuleuven.be; 3 Institute for Health Metrics & Evaluation, University of Washington, Seattle, WA 98195, USA; charles.jonscher@cet.co.uk; 4 Department of Radiology, UZ Gasthuisberg, Herestraat 49, 3000 Leuven, Belgium; chantal.vanongeval@uzleuven.be

**Keywords:** metastasis, cancer, epidemiology, lung, SEER

## Abstract

**Simple Summary:**

Cancer patients with synchronous lung metastasis (sLM) are recognized as an entity with poor survival, and no consensus exists about which patients may benefit from active treatment. The current study demonstrates disparities in the prevalence and prognosis of sLM by primary cancer type and clinicodemographic factors, based on the SEER database. These heterogeneities here lay a foundation for and call for the development of risk assessment and prognosis classification tools that drive clinical management.

**Abstract:**

The epidemiology and associated potential heterogeneity of synchronous lung metastasis (sLM) have not been reported at a population-based level. Cancer patients with valid information about sLM status in the Surveillance, Epidemiology, and End Results database were enrolled. The prevalence of sLM, with a 95% confidential interval, and median survival of sLM, with interquartile range, were calculated and compared by Chi-square analyses and log-rank tests by primary cancer type and clinicopathological factors. Furthermore, the risk factors of sLM development were identified by multivariate logistic regression. Among 1,672,265 enrolled cases, 3.3% cases were identified with sLM, with a median survival of 7 months. Heterogeneity in prevalence and prognosis in sLM was observed among different primary cancers, with the highest prevalence in main bronchus cancer and best survival in testis cancer. Higher prevalence and poorer prognosis were observed in the older population, male population, African American, patients with lower socioeconomic status, and cases with advanced T stage, N stage, or more malignant pathological characteristics. Race, age, T stage, N stage, metastasis to other sites, insurance status and marital status were associated with sLM development (*p* < 0.001). The current study highlights the heterogeneity of the prevalence and prognosis in patients with sLM.

## 1. Introduction

Cancer represents a great threat to public health worldwide, with an estimated 18.1 million new cases and 9.6 million cancer-related deaths globally in 2018 [1]. Approximately 90% of cancer-related deaths can be attributed to cancer metastasis, a major indication of treatment failure [2]. Early detection and active treatment of primary lesions may help reduce the risk of the development of metastatic disease [3,4]. The prognosis for metastasized cases is generally poor due to high tumor burden, inferior performance status, limited therapeutic options, and impairment of the involved organs’ function. The lungs are the third most common metastatic site for cancer, following the liver and bone [5]. Based on their organ mechanics, specifically vein drainage from the systemic circulation and their large area of micro-circulation, the lungs are exposed to the great risk of metastasis. Additionally, the organotropism is also attributable to the exosomes secreted by the primary cancer cells to prepare the premetastatic niche [6].

Great heterogeneity among metastatic lesions concerning tumor burden, treatment response, prognosis, and so on challenges the crude “yes-or-no” classification system for stratifying the metastasis status of cancer patients. For instance, a modified M stage by the organs being metastasized was proposed in pancreatic neuroendocrine tumor [7]. In another perspective of metastasis, extra heterogeneity also exists in terms of metastatic origin; that is, patients with metastatic lesions from different primary cancers show divergent disease characteristics, treatment responses, prognoses, and so on [8]. Cancer metastasis was conventionally believed to be incurable; however, such heterogeneities raise hope for selecting patients who may potentially benefit from the evolving therapeutics from palliative or best supportive cares towards active interventions or even curative attempts. Individualized recommendation of treatment protocol is based on the precise prognosis prediction and thus the selection of patients who are expected to benefit more from the treatment. For sLM, currently available treatment includes stereotactic body radiation therapy, surgery, and ablation, followed by an in-depth understanding of cancer biology [9]. However, the heterogeneity of seeding primary cancer cells which also drive the prognosis disparity remains largely unknown and the few studies are elaborating on prognostic stratification of patients with sLM. There are previous publications on relevant topics that mainly focus on the molecular mechanism of sLM formation, retrospective studies on the treatment of sLM, and epidemiological studies based on small sample size, or from only colorectal cancer or breast cancer [10,11]. In addition to the heterogeneity of metastasis caused by biological factors, the formation of metastasis may be also influenced by socioeconomic status (SES) [12].

The current study was designed to test the hypothetical disparities with regards to the prevalence and prognosis within sLM patients with different biological features of the primary tumors and different socioeconomic statuses across different cancer types or even sub-types within certain cancers. To achieve sufficient representativeness and generalizability, we explored the Surveillance, Epidemiology, and End Results (SEER) database to achieve estimations of the prevalence and prognosis of sLM. Point estimates of the prevalence along with 95% confidence interval and median survival, along with interquartile range, were used to compare risk of developing sLM and prognosis.

## 2. Materials and Methods

### 2.1. Eligible Patients

The SEER database, founded by the National Cancer Institute in 1973, currently enrolls cancer cases from 18 registries, covering about 28% of all the U.S. population, and an increasing number of variables were collected by the SEER database over time [13]. The metastatic status of the lungs was first available in 2010; thus, only cases between 1 January 2010 and 31 December 2016 (most updated) with valid synchronous lung metastasis (sLM) status were eligible for this study. sLM is most likely diagnosed by radiology exams, which, however, failed to discriminate multi-focal primary lung cancer (all nodules are primary lung cancer) from metastatic lung cancer (nodules are primary and metastatic). Further pathological and bioinformatic results for these nodules are currently unavailable for clear classification in the SEER database. Thus, to ensure the quality of the current study, the cases of primary lung cancer anatomically in the lungs were excluded. Additionally, bronchus cancer was enrolled to increase the generalizability of this study, since bronchus can be easily discriminated from primary lung cancer in lung tissue by radiology exams. Cases with a prior cancer history or with T stages such as T0, Tis, or Ta (for bladder cancer) were excluded. Only sLM cases with the diagnosis of their primary cancers by either histological or cytological exams were enrolled, and leukemia and lymphoma cases were excluded due to their diffuse nature.

### 2.2. Tumor Classification and Statistical Analyses

The primary cancer types were determined by the information of both organ site and histological diagnosis, except for sarcoma and melanoma, which are defined solely by histology [14,15]. Embryonal tumors include medulloblastoma, Wilms tumor, and neuroblastoma. The subgrouping of cancer types was determined for any particular cancers based on detailed clinical information. Age at diagnosis was categorized into five stratifications (0–18, 19–40, 41–60, 61–80 and 81+ years). The race here includes four classes: Caucasian, African American, other races, and unknown, based on the SEER database [16]. The patients are categorized by the AJCC 7th TNM stages [17].

The prevalence of sLM along with its 95% confidence interval and the ratio of sLM over all metastases were calculated, following the summary of the number of all cases, metastatic cases, and sLM cases by primary cancer (Table 1 and Table 2) [18]. The prognosis for sLM cases was depicted by median survival and interquartile range, estimated by the Kaplan–Meier method, and the survival difference was assessed by log-rank tests. Comparison by biological variables and socioeconomic factors was also performed. Here, due to the unavailability of individual-level income, and education information, county-level surrogates were adopted by linking the residing address to the 2013 Rural-Urban Continuum Codes from the United States Department of Agriculture [19]. Multivariate logistic regression adjusted for primary cancer site was performed to assess the relationship between clinical demographic factors and the occurrence of sLM patients. Statistical analyses were performed on R 3.6.0 (https://www.R-project.org/ (accessed on 8 January 2022)), with the survminer and survival package [20,21].

## 3. Results

### 3.1. Prevalence of Lung Metastasis

**Figure 1:**
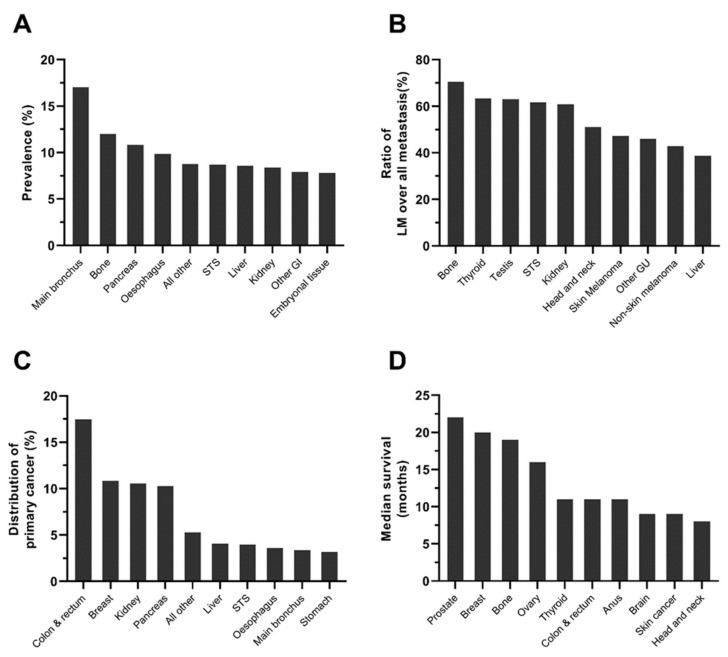
Prevalence and prognosis of sLM cases by primary cancer. (**A**) Prevalence of synchronous sLM by cancer type in all cancer patients (including metastatic and non-metastatic cancer patients); (**B**) Ratio of synchronous sLM by cancer type in patients with metastatic lesions; (**C**) Distribution of primary cancer in patients with sLM; (**D**) Median survival of cancer patients with sLM. Abbreviations: STS: soft tissue sarcoma; GI: gastrointestinal cancer; GU: genitourinary cancer.

1,672,265 cases were eligible for the current study, with 194,012 total metastatic cases and 55,193 sLM cases, which accounts for 3.30% (95% CI: 3.27–3.33%) of all cases and 28.45% of metastatic cases, respectively. The prevalence of sLM varies greatly across different cancer types (Table 1). Tumor types with the highest sLM prevalence were tumors originating from the main bronchus, 17.03% (95% CI: 16.33–17.74%), bone, 11.99% (95% CI: 11.10–12.88%), pancreas, 10.83% (95% CI: 10.57–11.10%), oesophagus, 9.85% (95% CI: 9.43–10.26%), and kidney, 8.37% (95% CI: 8.16–8.57%) (Table 1, Figure 1). Significant prevalence disparities were observed among different age groups, sexes, races, patients with different T or N stages, and patients with different socioeconomic statuses (insurance, marriage, income, residence type, and education) (Table 2, Supplementary Tables S1–S5). Of note, a counterintuitively higher sLM prevalence was observed in the T1 stage compared with T2 cases in oesophagus cancer (7.87% vs. 2.85%, *p* < 0.001), gastric cancer (4.03% vs. 2.14%, *p* < 0.001), extrahepatic biliary tract cancer (3.81% vs. 1.59%, *p* < 0.001), and CRC (3.01% vs. 0.79%, *p* < 0.001) ( Supplementary Table S4). Similarly, a significantly higher prevalence in N1 cases compared with N2 cases was observed in oesophagus cancer (12.59% vs. 6.70%, *p* < 0.001), gastric cancer (8.38% vs. 2.51%, *p* < 0.001), and CRC (6.73% vs. 6.42%, *p* < 0.001).

sLM is the major type of metastasis for metastatic cases originating from bone (70.57%), thyroid (63.28%), testis (63.04%), soft tissue (61.66%), kidney (60.88%), and head and neck (51.12%) (Table 1). In terms of the distribution of primary cancers, 17.48% of sLM cases originated from CRC, with 10.82% from the breast, 10.55% from the kidney, and 10.29% from the pancreas (Table 1). Demographic factors (race, age), clinical factors (T stage, N stage, bone metastasis, brain metastasis, liver metastasis), and socioeconomic factors (marital status, insurance status) are associated with the development of sLM, as revealed by multivariate logistic regression adjusted by primary cancer site (Table 3).

### 3.2. Survival Analysis

**Figure 2:**
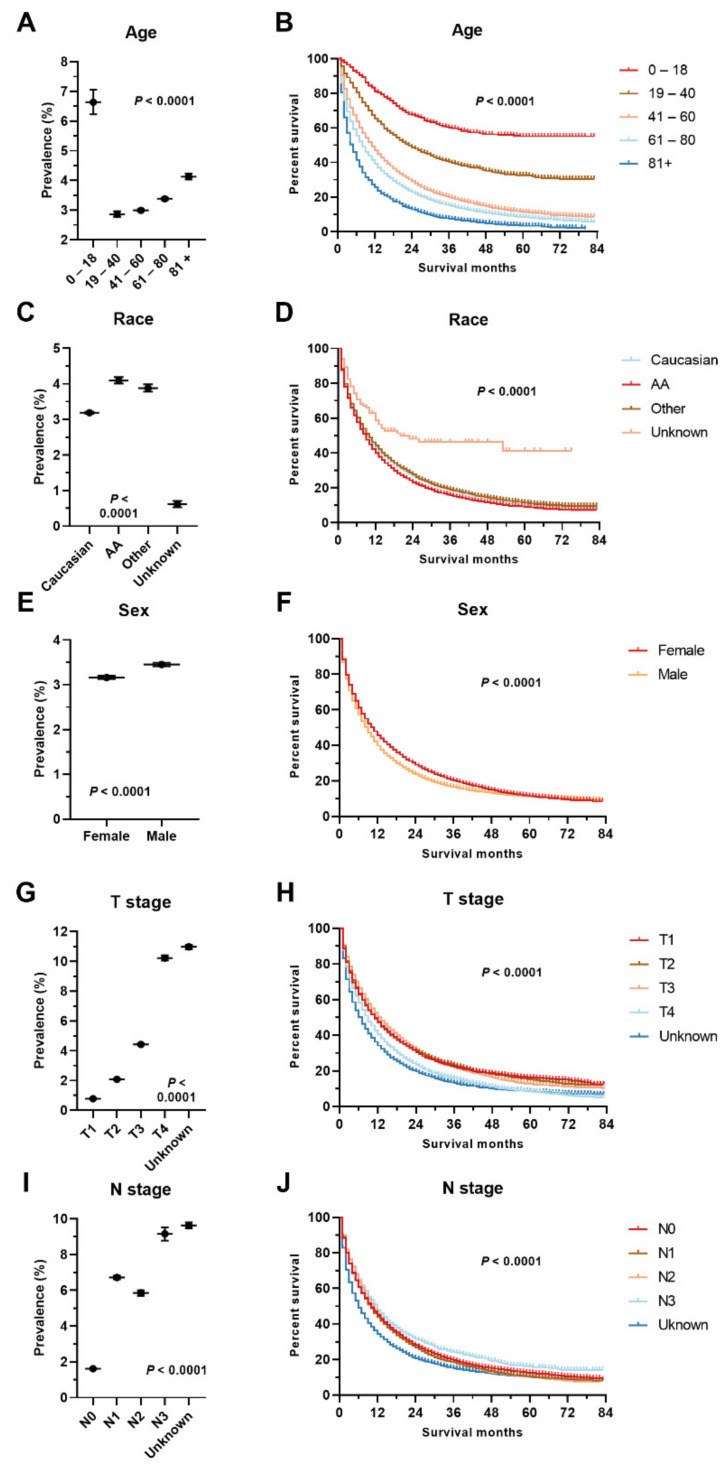
Disparities in prevalence and prognosis for sLM by age (**A**,**B**), race (**C**,**D**), sex (**E**,**F**), T stage (**G**,**H**), and N stage (**I**,**J**). Statistical tests were performed by Chi-square analyses and log-rank tests. Abbreviation: AA: African American.

**Figure 3:**
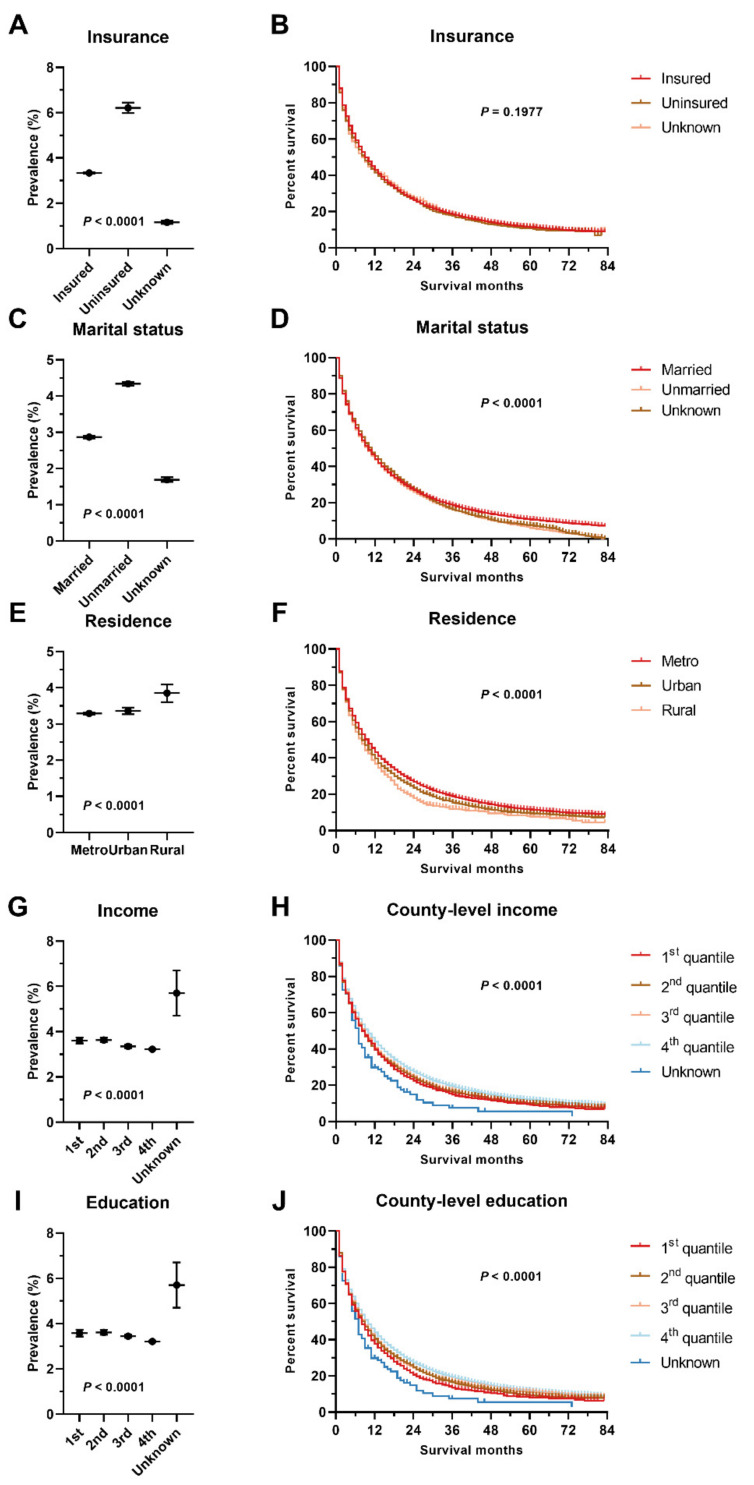
Disparities in prevalence and prognosis for sLM by insurance (**A**,**B**), marital status (**C**,**D**), residence type (**E**,**F**), county-level income (**G**,**H**), and county-level education (**I**,**J**). Statistical tests were performed by Chi-square analyses and log-rank tests.

The median survival and corresponding interquartile range for sLM cases by primary cancers are presented in Table 1. The median survival for general sLM cases is 7 months, with the best survival in testis cancer and embryonal tumors (median survival unreached), prostate cancer (22 months), breast cancer (20 months), bone tumor (19 months), ovary cancer (16 months), thyroid cancer (11 months), and CRC (10 months) (Table 1). Similarly, survival disparities can be observed in patients with sLM among different age groups, sexes, races, different T or N stages, and patients with different socioeconomic statuses (insurance, marriage, income, residence type, education, and unemployment) (Table 2, Supplementary Tables S1–S5). Better prognosis in patients with sLM was observed in females, other race, younger patients, patients who resided in richer or better-educated counties, metro or urban cities, insured patients, and married patients (Figure 2 and Figure 3). Consistent with the counterintuitive prevalence pattern in gastrointestinal cancers, a better prognosis was observed in the slightly advanced cases of these cases with sLM.

## 4. Discussion

The current study has reported for the first time the prevalence and prognosis of synchronous lung metastasis (sLM) by different primary cancer types, based on a population-based database with great generalizability and representativeness. Furthermore, subgroup analyses confirm hypothetical disparities in prevalence and prognosis inside subgroups by both biological factors and socioeconomic factors. These disparities here may highlight the heterogeneity of sLM, which calls for the development of clinical tools for risk stratification and prognosis classification.

The diverse prevalence and prognosis observed here may be biologically attributed to two aspects: microenvironment in lung tissue and primary cancer cell. The “soil,” or microenvironment, of lungs influences the formation of sLM, with smoking being verified as a risk factor by clinical retrospective studies and bench studies [22]. Similarly, a retrospective study of 567 pathological stage I, II, or III colorectal cancers showed that current smokers harbor a higher risk of sLM (hazard ratio = 2.72, 95% CI 1.18–6.25; *p* = 0.02) [23]. Smoking can promote the formation of sLM in colorectal cancer animal models [24]. In the B16-MO5 melanoma animal model, smoking exposure impairs the NK cell-dependent anti-tumor immunity, which can be resecured by activation through bone marrow-derived dendritic cells [25]. A case-control study with 87 female breast cancer patients with sLM and 174 controlled cases without sLM showed a relationship between active smoking and the development of sLM, which was further validated by an animal study [26,27]. Smoke-associated chronic inflammation in the lung microenvironment may promote growth of metastatic cancer cells from breast cancer in mice experiments and patients [28,29]. Interestingly, e-cigarettes also promote breast cancer cell growth and metastatic lung colonization, mediated by cross-talk with tumor-associated macrophages via CCL5 and VCAM-1 pathways in an animal study [30]. However, due to a lack of disease information about the intrinsic lung disease in the SEER database, we failed to verify these findings. Additionally, the development of clinical management of lung metastasis, including surgical techniques and post-treatment care, may help improve the prognosis and quality of life [31,32].

Besides the soil, the pattern of metastasis epitomizes the role of heterogeneous cancer biology. In the subgroup analyses, heterogeneities in tumor biology (tumor marker level, histology characteristics) are translated into disparities in prevalence and prognosis, indicating that involvement of a detailed molecular profile may boost more precise estimation, such as what we achieved in the prognostic classification of brain metastasis. Elevated tumor biomarker level, including AFP, CEA, PSA, and CA125, is usually associated with either higher tumor burden or increased malignancy and thus the intuitively higher prevalence of sLM and poorer survival. HER+ breast cancer is associated with a higher prevalence of sLM and liver metastasis in clinic, which is mediated by its switching effect of TGFβ from inhibiting cell proliferation to promoting cell migration [8,33]. Nottingham grade was believed to be predictive in cancer-specific survival and disease-free survival in breast cancer, and we also observed its predictive role in breast cancer sLM cases, with lower prevalence and better survival in patients with a lower Nottingham grade [34]. RLIP is associated with breast cancer growth and metastasis to the lungs, based on results from an animal study [35]. In line with a bench study, AFP is associated with an increased risk of developing sLM by up-regulation of metastasis-related proteins [36]. The disparity in the prevalence of sLM by fibrosis status can be partly explained by their baseline characteristics: compared with cirrhotic HCC cases, patients without cirrhosis generally present worse pathological grade, larger tumor, and more lymph node involvement [37]. Interestingly, sidedness of CRC also impacts the formation and prognosis of sLM, with lower prevalence but poorer prognosis in right CRC, which may be explained by a more extensive metastasis pattern, poorer differentiation, and a higher percentage of KRAS and/or BRAF mutation in right CRC [38]. Unlike the divergent role of RAS in colorectal cancer liver metastasis, RAS mutation is associated with a higher prevalence of metastasis in lung, brain, and bone, with hazard ratios of 1.5, 3.7, and 1.6, respectively [39,40]. Interestingly, we discovered a counterintuitively higher prevalence of sLM in the patients with T1 stage than in the cases with more advanced stages in esophagus cancer, gastric cancer, and CRC. This disproportional relationship also exists in the prevalence of liver metastasis and brain metastasis in most of the cancer types mentioned above [8,41]. A bioinformatics study in CRC identified a set of genetic mutations associated with the development of metastasis years before a clinically detectable primary lesion [42]. The epidemiology data here may represent clues for bench researchers, who may decode the molecular interplay underlying the clinical manifestations.

SES not only affects primary cancer diagnosis, treatment, and prognosis but modifies the risk of developing metastasis and alters its prognosis [43]. The higher sLM prevalence and poorer sLM prognosis in patients with lower SES status can be explained by the lower awareness of early diagnosis, delayed treatment, and poorer access to healthcare resources [44]. For example, married patients are associated with better survival due to a higher rate of surgery or radiotherapy in all cancer patients as well as sLM patients [45]. Furthermore, the first impression of lower SES in unmarried patients may lead to less intensive treatment from clinicians [46]. Lower income was reported to halt early diagnosis and sufficient and timely treatment, which can be restored by insurance [47,48,49,50,51]. In terms of racial disparity, a higher risk of sLM in African Americans and other races can be attributed to the lower insurance coverage in the population [52], advanced stage of primary cancer at diagnosis [53], less participation in cancer screening [54], and so on. These factors interplay with each other and contribute to the disparities here, and there is a pressing need to increase awareness of this disparity.

Besides highlighting the disparities of sLM, the current study also provides descriptive data about sLM epidemiology. The prevalence data here help estimate the disease burden for pharmaceutical companies, healthcare insurance systems, allocation of lung-directed treatment resources, and so on. As most of the current treatments are designed to target the characteristics of tumor of origin, rather than the metastatic niche, non-metastatic patients are the most frequently enrolled cases and the prevalence data here may help estimate how many patients are needed during initial enrollment, and more importantly, this estimation can be more precise after considering the subgroup disparities. Also, the survival data here may help estimate the sample size for clinical trials for a given expected survival difference.

Despite novel findings in the current study, it harbors the following limitations. Firstly, the SEER database only provides qualitative information about the sLM status, which disenables quantitative analyses by tumor number and tumor size. Secondly, the diagnostic method for the sLM remained unreported, and the heterogeneity in the detecting sensitivity may have biased the prevalence data here. Thirdly, the co-existing lung disease status and the detailed genetic information of primary cancer (such as RAS mutation status in CRC, BRAF mutation in melanoma, and so on) remained unknown, which disenabled more precise epidemiological data on sLM.

## 5. Conclusions

The current study reports the prevalence and prognosis of sLM in a pan-cancer manner based on a population-based database with great representativeness. These data may (1) demonstrate the heterogeneity of sLM by providing epidemiological evidence and (2) provide descriptive epidemiology data for estimating sLM burden for the pharmaceutic industry and policymakers, decision-making concerning lung surveillance or screening, and designing clinical trials by providing data to balance the prevalence and survival disparities.

## Figures and Tables

**Table 1:** The number of all cases, metastatic cases, and cases with synchronous lung metastasis and their corresponding prevalence, distribution, and median survival with interquartile range by cancer type.

Categories	No. of Cases			Prevalence ^a^	Ratio	Distribution ^c^	Survival ^d^
	All	Metastasis	sLM	sLM	sLM/Metastasis ^b^		
All	1,672,265	194,012	55,193	3.30% (3.27–3.33%)	28.45%	100.00%	7 (2–22)
Brain	27,485	209	17	0.06% (0.03–0.09%)	8.13%	0.03%	9 (3–23)
Head and neck	74,897	3079	1574	2.10% (2.00–2.20%)	51.12%	2.85%	8 (3–20)
Thyroid	78,003	1574	996	1.28% (1.20–1.36%)	63.28%	1.80%	11 (2–79)
Pathology							
Solitary	45,450	782	516	1.14% (1.04–1.23%) ***	65.98%	0.93%	9 (2–70) ***
Multifocal	30,707	514	309	1.01% (0.89–1.12%)	60.12%	0.56%	45 (6–NA)
Unknown	1846	278	171	9.26% (7.94–10.59%)	61.51%	0.31%	4 (1–13)
All breast	358,649	18,819	5972	1.67% (1.62–1.71%)	31.73%	10.82%	20 (5–47)
Molecular subtype							
Her2−/HR+	15,831	1564	575	3.63% (3.34–3.92%) ***	36.76%	1.04%	20 (4–52) ***
Her2+/HR+	37,204	2905	907	2.44% (2.28–2.59%)	31.22%	1.64%	32 (10–NA)
Her2+/HR−	245,301	9878	2794	1.14% (1.10–1.18%)	28.29%	5.06%	27 (8–52)
Triple negative	37,359	2303	944	2.53% (2.37–2.69%)	40.99%	1.71%	10 (3–19)
Unknown	22,954	2169	752	3.28% (3.05–3.51%)	34.67%	1.36%	8 (1–30)
BR grade							
Low	82,105	1245	302	0.37% (0.33–0.41%) ***	24.26%	0.55%	34 (10–65) ***
Medium	140,840	5221	1474	1.05% (0.99–1.10%)	28.23%	2.67%	28 (10–61)
High	95,779	5413	1949	2.03% (1.95–2.12%)	36.01%	3.53%	18 (5–43)
Unknown	39,925	6940	2247	5.63% (5.40–5.85%)	32.38%	4.07%	15 (2–40)
Main bronchus	10,878	6848	1853	17.03% (16.33–17.74%)	27.06%	3.36%	3 (1–10)
Oesophagus	20,068	6576	1976	9.85% (9.43–10.26%)	30.05%	3.58%	4 (1–10)
Stomach	31,825	11,559	1756	5.52% (5.27–5.77%)	15.19%	3.18%	3 (1–9)
Liver ^e^	26,267	5793	2245	8.55% (8.21–8.88%)	38.75%	4.07%	2 (1–6)
AFP level							
Elevated	11,959	2598	1034	8.65% (8.14–9.15%) ***	39.80%	1.87%	2 (1–5) ***
Normal	6713	1191	445	6.63% (6.03–7.22%)	37.36%	0.81%	3 (1–8)
Borderline	59	10	2	NA	20.00%	0.00%	8 (8–8)
Unknown	7536	1324	494	6.56% (6.00–7.11%)	37.31%	0.90%	2 (1–8)
Fibrosis grade							
None to moderate	1923	277	101	5.25% (4.26–6.25%) ***	36.46%	0.18%	4 (2–9) ***
Severe or cirrhotic	4178	524	186	4.45% (3.83–5.08%)	35.50%	0.34%	2 (0–6)
Unknown	20,166	4992	1958	9.71% (9.30–10.12%)	39.22%	3.55%	2 (1–6)
Extrahepatic biliary tract	14,238	4727	876	6.15% (5.76–6.55%)	18.53%	1.59%	3 (1–8)
Pancreas	52,442	26,544	5682	10.83% (10.57–11.10%)	21.41%	10.29%	3 (1–7)
Tumor site							
Head of pancreas	25,958	9343	1766	6.80% (6.50–7.11%) ***	18.90%	3.20%	3 (1–9) ***
Body of pancreas	7272	4215	919	12.64% (11.87–13.40%)	21.80%	1.67%	3 (1–9)
Tail of pancreas	8186	5663	1240	15.15% (14.37–15.92%)	21.90%	2.25%	2 (1–6)
Unspecified pancreas	11,026	7323	1757	15.94% (15.25–16.62%)	23.99%	3.18%	2 (1–6)
Small intestine	9628	2584	239	2.48% (2.17–2.79%)	9.25%	0.43%	8 (2–22)
Colon & rectum	186,539	37,739	9645	5.17% (5.07–5.27%)	25.56%	17.48%	11 (3–25)
Tumor site							
Right colon	72,383	13,857	2777	3.84% (3.70–3.98%) ***	20.04%	5.03%	8 (2–21) ***
Left colon	64,014	13,773	3370	5.26% (5.09–5.44%)	24.47%	6.11%	13 (3–28)
Unspecified colon	4727	2549	746	15.78% (14.74–16.82%)	29.27%	1.35%	3 (1–12)
Rectum	45,415	7560	2752	6.06% (5.84–6.28%)	36.40%	4.99%	15 (5–29)
CEA level							
CEA elevated	51,520	21,670	5987	11.62% (11.34–11.90%) ***	27.63%	10.85%	11 (3–24) ***
CEA normal	54,991	4642	840	1.53% (1.43–1.63%)	18.10%	1.52%	20 (7–38)
CEA borderline	547	69	11	2.01% (0.83–3.19%)	15.94%	0.02%	11 (3–27)
CEA unknown	38,692	1546	178	0.46% (0.39–0.53%)	11.51%	0.32%	14 (4–50)
Perineural Invasion							
Yes	16,415	5333	944	5.75% (5.39–6.11%) ***	17.70%	1.71%	18 (6–34) ***
No	124,544	15,576	3332	2.68% (2.59–2.76%)	21.39%	6.04%	15 (5–31)
Unknown	45,580	16,830	5369	11.78% (11.48–12.08%)	31.90%	9.73%	8 (2–21)
Anus	9405	695	183	1.95% (1.67–2.22%)	26.33%	0.33%	11 (5–23)
Other GI	8611	3222	680	7.90% (7.33–8.47%)	21.10%	1.23%	2 (0–7)
Kidney	69,605	9564	5823	8.37% (8.16–8.57%)	60.88%	10.55%	8 (2–22)
Fuhrman grade							
I	6144	131	81	1.32% (1.03–1.60%) ***	61.83%	0.15%	12 (4–61) ***
II	28,826	906	511	1.77% (1.62–1.93%)	56.40%	0.93%	20 (7–44)
III	16,496	1624	956	5.80% (5.44–6.15%)	58.87%	1.73%	17 (6–40)
IV	4832	1569	1010	20.90% (19.76–22.05%)	64.37%	1.83%	11 (5–29)
Unknown	13,307	5334	3265	24.54% (23.80–25.27%)	61.21%	5.92%	4 (2–12)
Bladder	41,668	3348	1215	2.92% (2.75–3.08%)	36.29%	2.20%	4 (1–11)
Pathological grade							
Low	3231	61	27	0.84% (0.52–1.15%) ***	44.26%	0.05%	4 (2–15) ***
High	32,596	2322	813	2.49% (2.32–2.66%)	35.01%	1.47%	4 (2–11)
Unknown	5841	965	375	6.42% (5.79–7.05%)	38.86%	0.68%	3 (1–9)
Prostate	309,918	15,735	1339	0.43% (0.41–0.46%)	8.51%	2.43%	22 (8–63)
PSA level							
1st quantile	67,653	588	81	0.12% (0.09–0.15%) ***	13.78%	0.15%	11 (5–32) ***
2nd quantile	64,654	317	28	0.04% (0.03–0.06%)	8.83%	0.05%	14 (6–42)
3rd quantile	64,571	685	46	0.07% (0.05–0.09%)	6.72%	0.08%	22 (11–NA)
4th quantile	64,103	5332	373	0.58% (0.52–0.64%)	7.00%	0.68%	27 (9–72)
Unknown	48,937	8813	811	1.66% (1.54–1.77%)	9.20%	1.47%	20 (9–53)
Testis	15,881	1791	1129	7.11% (6.71–7.51%)	63.04%	2.05%	NA (20–NA)
Other GU	8718	1152	529	6.07% (5.57–6.57%)	45.92%	0.96%	5 (2–12)
Ovary	29,789	7790	1696	5.69% (5.43–5.96%)	21.77%	3.07%	16 (2–36)
CA125 level							
Elevated	20,383	6164	1343	6.59% (6.25–6.93%) ***	21.79%	2.43%	18 (3–37) ***
Normal	2761	176	29	1.05% (0.67–1.43%)	16.48%	0.05%	12 (4–25)
Borderline	36	4	0	NA	0.00%	0.00%	NA
Unknown	6609	1446	324	4.90% (4.38–5.42%)	22.41%	0.59%	7 (1–27)
Uterus	73,342	4206	1232	1.68% (1.59–1.77%)	29.29%	2.23%	8 (2–22)
Cervix	20,658	2712	844	4.09% (3.82–4.36%)	31.12%	1.53%	7 (3–17)
Other GYN	10,514	1355	457	4.35% (3.96–4.74%)	33.73%	0.83%	5 (1–23)
Bone tumor	5079	863	609	11.99% (11.10–12.88%)	70.57%	1.10%	19 (8–NA)
STS	25,093	3537	2181	8.69% (8.34–9.04%)	61.66%	3.95%	8 (2–23)
Skin Melanoma	107,287	2256	1065	0.99% (0.93–1.05%)	47.21%	1.93%	6 (2–20)
Ulceration							
Yes	13,441	619	268	1.99% (1.76–2.23%) ***	43.30%	0.49%	7 (3–22) ***
No	89,168	567	235	0.26% (0.23–0.30%)	41.45%	0.43%	11 (4–39)
Unknown	4678	1070	562	12.01% (11.08–12.95%)	52.52%	1.02%	4 (1–13)
Non-skin melanoma	3084	247	106	3.44% (2.79–4.08%)	42.91%	0.19%	6 (3–14)
Skin cancer	5345	203	37	0.69% (0.47–0.91%)	18.23%	0.07%	9 (6–18)
Embryonal tumors	4281	877	334	7.80% (7.00–8.61%)	38.08%	0.61%	NA (19–NA)
All other	33,068	8408	2903	8.78% (8.47–9.08%)	34.53%	5.26%	5 (1–20)

^a^ Prevalence was only calculated in categories with more than 5 sLM cases. ^b^ The ratios here represent the percentage of synchronous lung metastasis cases over all metastatic cases. ^c^ These data represent the percentage of synchronous lung metastasis from a specific site of origin over all lung metastasis cases. For instance, 0.03% of synchronous lung metastases originate from the brain. ^d^ Survival data (in months) are shown as the median survival and interquartile range in cases with synchronous lung metastasis at diagnosis. ^e^ Liver cancer here includes hepatocellular carcinoma and intrahepatic cholangiocarcinoma. Abbreviations: sLM: synchronous lung metastasis; HER-2: Human epidermal growth factor receptor-2; HR: hormone receptor; BR grade: Bloom–Richardson grade; AFP: alpha-fetoprotein; GI: gastrointestinal cancer; CRC: colorectal cancer; CEA: carcinoembryonic antigen; GU: genitourinary cancer; PSA: prostate specific antigen; CA125: cancer antigen 125; GYN: gynaecologic cancer; STS: soft-tissue sarcoma; NA: non-applicable; ***: *p* < 0.001 for intragroup survival comparison by Chi-square tests or log-rank tests.

**Table 2:** Number of all cases, metastatic cases and cases with synchronous lung metastasis and their corresponding prevalence, distribution, and median survival with interquartile range by clinicodemographic variables.

Categories	Number of Cases			Prevalence ^a^	Ratio	Distribution ^c^	Survival ^d^
	All	Metastasis	sLM	sLM	sLM/Metastasis ^b^		
Year of diagnosis							
2010	232,553	25,223	6776	2.91% (2.85–2.98%) ***	26.86%	12.28%	7 (2–22) ***
2011	236,568	25,572	7144	3.02% (2.95–3.09%)	27.94%	12.94%	7 (2–22)
2012	234,008	26,323	7504	3.21% (3.14–3.28%)	28.51%	13.60%	7 (2–21)
2013	234,946	27,393	7878	3.35% (3.28–3.43%)	28.76%	14.27%	7 (2–23)
2014	238,679	28,404	8172	3.42% (3.35–3.50%)	28.77%	14.81%	7 (2–23)
2015	245,850	29,658	8544	3.48% (3.40–3.55%)	28.81%	15.48%	7 (2–22)
2016	249,661	31,439	9175	3.67% (3.60–3.75%)	29.18%	16.62%	8 (2–NA)
Sex							
Female	873,620	94,869	27,642	3.16% (3.13–3.20%) ***	29.14%	50.08%	8 (2–25) ***
Male	798,645	99,143	27,551	3.45% (3.41–3.49%)	27.79%	49.92%	7 (2–20)
Race							
Caucasian	1,323,660	149,175	42,188	3.19% (3.16–3.22%) ***	28.28%	76.44%	7 (2–23) ***
African American	187,441	27,368	7687	4.10% (4.01–4.19%)	28.09%	13.93%	7 (2–20)
Other	132,299	16,772	5138	3.88% (3.78–3.99%)	30.63%	9.31%	8 (2–24)
Unknown	28,865	697	180	0.62% (0.53–0.71%)	25.82%	0.33%	14 (4–NA)
Age group							
0–18	13,939	1873	926	6.64% (6.23–7.06%) ***	49.44%	1.68%	NA (17–NA) ***
19–40	115,626	9374	3311	2.86% (2.77–2.96%)	35.32%	6.00%	21 (7–NA)
41–60	597,058	63,954	17,863	2.99% (2.95–3.04%)	27.93%	32.36%	9 (3–26)
61–80	794,720	96,147	26,863	3.38% (3.34–3.42%)	27.94%	48.67%	6 (1–19)
81+	150,922	22,664	6230	4.13% (4.03–4.23%)	27.49%	11.29%	3 (1–10)
T stage							
T1	715,832	20,927	5618	0.78% (0.76–0.81%) ***	26.85%	10.18%	9 (2–27) ***
T2	403,599	30,559	8392	2.08% (2.04–2.12%)	27.46%	15.20%	9 (2–29)
T3	285,021	48,304	12,631	4.43% (4.36–4.51%)	26.15%	22.89%	11 (3–29)
T4	110,768	39,789	11,325	10.22% (10.05–10.40%)	28.46%	20.52%	7 (2–20)
Unknown	157,045	54,433	17,227	10.97% (10.81–11.12%)	31.65%	31.21%	4 (1–15)
N stage							
N0	1,184,206	72,679	19,196	1.62% (1.60–1.64%) ***	26.41%	34.78%	8 (2–25) ***
N1	255,900	59,633	17,193	6.72% (6.62–6.82%)	28.83%	31.15%	8 (2–23)
N2	90,865	21,136	5313	5.85% (5.69–6.00%)	25.14%	9.63%	9 (2–24)
N3	23,729	7065	2173	9.16% (8.79–9.52%)	30.76%	3.94%	10 (3–30)
Unknown	117,565	33,499	11,318	9.63% (9.46–9.80%)	33.79%	20.51%	4 (1–15)
Insurance status							
Insured	1,540,367	181,774	51,501	3.34% (3.32–3.37%) ***	28.33%	93.31%	7 (2–23) ^ns^
Uninsured	42,868	8546	2663	6.21% (5.98–6.44%)	31.16%	4.82%	6 (1–21)
Unknown	89,030	3692	1029	1.16% (1.09–1.23%)	27.87%	1.86%	5 (1–22)
Marital status							
Married	911,531	97,059	26,141	2.87% (2.83–2.90%) ***	26.93%	47.36%	8 (2–23) ***
Unmarried	610,219	87,716	26,503	4.34% (4.29–4.39%)	30.21%	48.02%	6 (2–21)
Unknown	150,515	9237	2549	1.69% (1.63–1.76%)	27.60%	4.62%	7 (2–23)
County-level income							
1st quantile	80,355	10,542	2900	3.61% (3.48–3.74%) ***	27.51%	5.25%	6 (2–19) ***
2nd quantile	162,810	20,689	5917	3.63% (3.54–3.73%)	28.60%	10.72%	6 (2–20)
3rd quantile	223,745	26,546	7504	3.35% (3.28–3.43%)	28.27%	13.60%	7 (2–21)
4th quantile	1,203,286	135,898	38,754	3.22% (3.19–3.25%)	28.52%	70.22%	8 (2–23)
Unknown	2069	337	118	5.70% (4.70–6.70%)	35.01%	0.21%	5 (1–13)
County-level education							
1st quantile	58,086	7592	2078	3.58% (3.43–3.73%) ***	27.37%	3.76%	6 (2–18) ***
2nd quantile	125,184	15,693	4524	3.61% (3.51–3.72%)	28.83%	8.20%	6 (2–20)
3rd quantile	301,780	36,451	10,382	3.44% (3.38–3.51%)	28.48%	18.81%	7 (2–20)
4th quantile	1,185,146	133,939	38,091	3.21% (3.18–3.25%)	28.44%	69.01%	8 (2–24)
Unknown	2069	337	118	5.70% (4.70–6.70%)	35.01%	0.21%	5 (1–13)
Residence							
Metro	1,489,707	171,907	48,948	3.29% (3.26–3.31%) ***	28.47%	88.69%	7 (2–23) ***
Urban	159,003	19,010	5346	3.36% (3.27–3.45%)	28.12%	9.69%	7 (2–20)
Rural	23,351	3086	898	3.85% (3.60–4.09%)	29.10%	1.63%	6 (2–17)
Unknown	204	9	1	NA	11.11%	0.00%	73 (73–73)

^a^ Prevalence was only calculated in categories with more than 5 sLM cases. ^b^ The ratios here represent the percentage of synchronous lung metastasis cases over all metastatic cases. ^c^ These data represent the percentage of synchronous lung metastasis from a specific group over all lung metastasis cases. For instance, 12.28% of synchronous lung metastases were diagnosed in 2010. ^d^ Survival data (in months) are shown as the median survival with interquartile range by Kaplan–Meier analyses in cases with synchronous lung metastasis at diagnosis, and the statistical tests were performed by log-rank tests. Abbreviation: NA: non-applicable; sLM: synchronous lung metastasis. ^ns^: non-significant; ***: *p* < 0.001% for intragroup survival comparison by Chi-square tests or log-rank tests.

**Table 3:** Multivariate logistic regression of occurrence of synchronous lung metastasis adjusted by primary cancer site.

Categories	OR	95% CI	*p*-Value	Categories	OR	95% CI	*p*-Value
Sex				Liver metastasis			
Female	Reference			Yes	Reference		
Male	1.01	(0.98–1.03)	ns	No	0.16	(0.16–0.17)	***
Race				Unknown	0.83	(0.75–0.92)	***
Caucasian	Reference			NA	0.71	(0.45–1.14)	ns
African American	1.17	(1.14–1.21)	***	Insurance			
Other	1.15	(1.11–1.19)	***	Insured	Reference		
Unknown	0.46	(0.39–0.53)	***	Uninsured	1.27	(1.21–1.33)	***
Age group				Unknown	0.68	(0.64–0.74)	***
0–18	Reference			Marital status			
19–40	0.70	(0.63–0.77)	***	Married	Reference		
41–60	0.82	(0.74–0.90)	***	Unmarried	1.18	(1.16–1.20)	***
61–80	1.01	(0.92–1.12)	ns	Unknown	0.90	(0.86–0.95)	***
81+	1.10	(0.99–1.21)	ns	County-level income			
T stage				1st quantile	Reference		
T1	Reference			2nd quantile	1.02	(0.96–1.08)	ns
T2	2.37	(2.28–2.46)	***	3rd quantile	0.99	(0.93–1.04)	ns
T3	3.10	(2.99–3.21)	***	4th quantile	0.98	(0.93–1.04)	ns
T4	5.80	(5.58–6.03)	***	Unknown	NA	NA	NA
TX	5.43	(5.22–5.64)	***	County-level education			
N stage				1st quantile	Reference		
N0	Reference			2nd quantile	1.03	(0.96–1.10)	ns
N1	2.20	(2.14–2.25)	***	3rd quantile	1.02	(0.95–1.08)	ns
N2	1.84	(1.77–1.92)	***	4th quantile	0.99	(0.93–1.06)	ns
N3	2.74	(2.59–2.91)	***	Unknown	1.32	(1.05–1.67)	*
NX	1.71	(1.65–1.77)	***	Residence			
Bone metastasis				Metro	Reference		
Yes	Reference			Urban	0.99	(0.95–1.03)	ns
No	0.17	(0.17–0.18)	***	Rural	1.06	(0.97–1.16)	ns
Unknown	0.53	(0.48–0.60)	***	Unknown	0.34	(0.05–2.50)	ns
NA	0.73	(0.29–1.85)	ns				
Brain metastasis							
Yes	Reference						
No	0.17	(0.16–0.18)	***				
Unknown	0.44	(0.39–0.49)	***				
NA	0.65	(0.26–1.64)	ns				

Abbreviations: OR: odds ratio; 95% CI: 95% confidence interval; HR: hazard ratio; NA: non-applicable, ns: non-significant; *: <0.05; ***: <0.001 for comparison with reference group.

## Data Availability

All data here are publicly available in the SEER database (https://seer.cancer.gov/ (accessed on 12 December 2021)).

## Supplementary Materials

The following supporting information can be downloaded at: https://www.mdpi.com/article/10.3390/cancers14071608/s1, Supplementary Table S1. Number of cases with synchronous lung metastasis and all cases, prevalence of synchronous lung metastasis and median survival with interquartile range by cancer type and race or sex; Supplementary Table S2. Number of cases with synchronous lung metastasis and all cases, prevalence of synchronous lung metastasis and median survival with interquartile range by cancer type and age or county-level income; Supplementary Table S3. Number of cases with synchronous lung metastasis and all cases, prevalence of synchronous lung metastasis and median survival with interquartile range by cancer type and county-level education or county-level residence type; Supplementary Table S4. Number of cases with synchronous lung metastasis and all cases, prevalence of synchronous lung metastasis and median survival with interquartile range by cancer type and T stage or N stage; Supplementary Table S5. Number of cases with synchronous lung metastasis and all cases, prevalence of synchronous lung metastasis and median survival with interquartile range by cancer type and insurance status or marital status.
