# Supplementary material for: Heterogeneity of Synchronous Lung Metastasis Calls for Risk Stratification and Prognostic Classification: Evidence from a Population-Based Database
Source: Cancers (Basel). 2022 Mar 22;14(7):1608. doi: 10.3390/cancers14071608 (PMC8996888; doi:10.3390/cancers14071608)
Supplement: Supplementary file 1 [file cancers-14-01608-s001.zip › cancers-1641348-supplementary.pdf]

Supplementary Table S1. Number of cases with synchronous lung metastasis and all cases, prevalence of synchronous lung metastasis and median survival with interquartile range by cancer type and race or sex.

Categories	Race	No. of sLM (All)	Prevalence [a]	Survival [b]	Sex	No. of sLM (All)	Prevalence [a]	Survival [b]
Brain	Caucasian	14 (23,690)	0.06% (0.03%-0.09%) [ns]	10 (3-23) [*]	Female	8 (11,946)	0.07% (0.02%-0.11%) [ns]	3 (2-10) [*]
	AA	1 (1,810)	NA	NA (NA-NA)	Male	9 (15,539)	0.06% (0.02%-0.10%)	18 (9-32)
	Other	2 (1,789)	NA	2 (1-3)				
	Unknown	0 (196)	NA	NA				
Head and neck	Caucasian	1,168 (61,067)	1.91% (1.80%-2.02%) [***]	8 (3-20) [***]	Female	354 (19,683)	1.80% (1.61%-1.98%) [***]	9 (3-24) [ns]
	AA	264 (7,628)	3.46% (3.05%-3.87%)	7 (3-17)	Male	1,220 (55,214)	2.21% (2.09%-2.33%)	8 (3-20)
	Other	138 (5,392)	2.56% (2.14%-2.98%)	13 (5-32)				
	Unknown	4 (810)	NA	16.5 (10.5-24)				
Thyroid	Caucasian	748 (62,161)	1.20% (1.12%-1.29%) [***]	10 (2-79) [ns]	Female	529 (59,891)	0.88% (0.81%-0.96%) [***]	9 (2-NA) [ns]
	AA	93 (5,546)	1.68% (1.34%-2.01%)	14 (1-NA)	Male	467 (18,112)	2.58% (2.35%-2.81%)	11 (2-79)
	Other	150 (8,989)	1.67% (1.40%-1.93%)	13 (2-NA)				
	Unknown	5 (1,307)	0.38% (0.05%-0.72%)	10 (5-NA)				
Breast	Caucasian	4,261 (280,603)	1.52% (1.47%-1.56%) [***]	20 (5-49) [***]	Female	5,886 (356,039)	1.65% (1.61%-1.70%) [***]	20 (5-47) [ns]
	AA	1,155 (40,698)	2.84% (2.68%-3.00%)	15 (4-39)	Male	86 (2,610)	3.30% (2.61%-3.98%)	24 (5-64)
	Other	527 (34,338)	1.53% (1.40%-1.66%)	27 (8-53)				
	Unknown	29 (3,010)	0.96% (0.61%-1.31%)	53 (53-NA)				
Main bronchus	Caucasian	1444 (8,875)	16.27% (15.50%-17.04%) [***]	3 (1-10) [ns]	Female	852 (4,881)	17.46% (16.39%-18.52%) [ns]	4 (1-11) [***]
	AA	262 (1,394)	18.79% (16.74%-20.85%)	4 (1-12)	Male	1,001 (5,997)	16.69% (15.75%-17.64%)	3 (1-9)
	Other	145 (587)	24.70% (21.21%-28.19%)	5 (1-13)				
	Unknown	2 (22)	NA	3 (3-3)				
Oesophagus	Caucasian	1,589 (16,847)	9.43% (8.99%-9.87%) [***]	4 (1-10) [ns]	Female	365 (4,101)	8.90% (8.03%-9.77%) [*]	4 (1-10) [ns]
	AA	271 (2,007)	13.50% (12.01%-15.00%)	3 (1-7)	Male	1,611 (15,967)	10.09% (9.62%-10.56%)	4 (1-9)
	Other	112 (1,133)	9.89% (8.15%-11.62%)	5 (2-11)				
	Unknown	4 (81)	NA	6.5 (1.5-10.5)				
Stomach	Caucasian	1,293 (22,378)	5.78% (5.47%-6.08%) [***]	4 (1-9) [ns]	Female	568 (12,065)	4.71% (4.33%-5.09%) [***]	3 (1-8) [ns]
	AA	220 (4,149)	5.30% (4.62%-5.98%)	2 (1-8)	Male	1,188 (19,760)	6.01% (5.68%-6.34%)	3 (1-10)
	Other	235 (5,047)	4.66% (4.07%-5.24%)	3 (1-9)				
	Unknown	8 (251)	3.19% (1.01%-5.36%)	4 (1-NA)				
Liver [c]	Caucasian	1,556 (18,396)	8.46% (8.06%-8.86%) [ns]	2 (1-7) [ns]	Female	652 (7,596)	8.58% (7.95%-9.21%) [ns]	3 (1-8) [***]
	AA	303 (3,477)	8.71% (7.78%-9.65%)	2 (0-5)	Male	1,593 (18,671)	8.53% (8.13%-8.93%)	2 (1-5)
	Other	377 (4,258)	8.85% (8.00%-9.71%)	3 (1-6)				
	Unknown	9 (136)	6.62% (2.44%-10.80%)	4 (2-7)				
Extrahepatic biliary tract	Caucasian	656 (10,733)	6.11% (5.66%-6.57%) [ns]	3 (1-8) [ns]	Female	478 (7,828)	6.11% (5.58%-6.64%) [ns]	3 (1-9) [ns]
	AA	102 (1,568)	6.51% (5.28%-7.73%)	3 (1-7)	Male	398 (6,410)	6.21% (5.62%-6.80%)	3 (1-7)
	Other	114 (1,874)	6.08% (5.00%-7.17%)	4 (1-10)				
	Unknown	4 (63)	NA	2 (2-5)				
Pancreas	Caucasian	4,469 (41,197)	10.85% (10.55%-11.15%) [ns]	3 (1-8) [ns]	Female	2,777 (25,288)	10.98% (10.60%-11.37%) [ns]	3 (1-8) [***]
	AA	697 (6,623)	10.52% (9.78%-11.26%)	2 (1-7)	Male	2,905 (27,154)	10.70% (10.33%-11.07%)	2 (1-7)
	Other	504 (4,449)	11.33% (10.40%-12.26%)	2 (1-7)				
	Unknown	12 (173)	6.94% (3.15%-10.72%)	2 (1-6)				
Small intestine	Caucasian	169 (7,325)	2.31% (1.96%-2.65%) [ns]	8 (2-22) [ns]	Female	127 (4,708)	2.70% (2.23%-3.16%) [ns]	8 (2-27) [ns]
	AA	56 (1,753)	3.19% (2.37%-4.02%)	7 (2-14)	Male	112 (4,920)	2.28% (1.86%-2.69%)	8 (1-17)
	Other	13 (438)	2.97% (1.38%-4.56%)	8 (1-35)				
	Unknown	1 (112)	NA	NA (NA-NA)				
Colon & rectum	Caucasian	7,055 (142,974)	4.93% (4.82%-5.05%) [***]	12 (3-26) [***]	Female	4,358 (88,810)	4.91% (4.77%-5.05%) [***]	11 (3-26) [ns]
	AA	1,569 (23,428)	6.70% (6.38%-7.02%)	10 (3-22)	Male	5,287 (97,729)	5.41% (5.27%-5.55%)	12 (3-25)
	Other	999 (18,370)	5.44% (5.11%-5.77%)	11 (3-25)				
	Unknown	22 (1,767)	1.25% (0.73%-1.76%)	12 (6-23)				
Anus	Caucasian	147 (7,948)	1.85% (1.55%-2.15%) [***]	11 (5-23) [ns]	Female	103 (5,991)	1.72% (1.39%-2.05%) [*]	13 (5-22) [ns]
	AA	20 (1,091)	1.83% (1.04%-2.63%)	10 (6-28)	Male	80 (3,414)	2.34% (1.84%-2.85%)	11 (5-28)
	Other	14 (289)	4.84% (2.37%-7.32%)	6 (4-16)				
	Unknown	2 (77)	NA	13 (13-13)				
Other GI	Caucasian	512 (6,949)	7.37% (6.75%-7.98%) [***]	2 (0-7) [ns]	Female	297 (4,632)	6.41% (5.71%-7.12%) [***]	2 (0-6) [ns]
	AA	89 (920)	9.67% (7.76%-11.58%)	2 (0-6)	Male	383 (3,979)	9.63% (8.71%-10.54%)	2 (0-7)
	Other	78 (645)	12.09% (9.58%-14.61%)	2 (1-5)				
	Unknown	1 (97)	NA	NA (NA-NA)				
Kidney	Caucasian	4,773 (56,080)	8.51% (8.28%-8.74%) [***]	8 (2-22) [***]	Female	1,727 (25,340)	6.82% (6.51%-7.13%) [***]	7 (2-19) [***]
	AA	552 (8,225)	6.71% (6.17%-7.25%)	5 (2-13)	Male	4,096 (44,265)	9.25% (8.98%-9.52%)	8 (3-23)
	Other	482 (4,618)	10.44% (9.56%-11.32%)	9 (3-26)				
	Unknown	16 (682)	2.35% (1.21%-3.48%)	NA (4-NA)				
Bladder	Caucasian	1,019 (36,085)	2.82% (2.65%-2.99%) [***]	4 (1-11) [ns]	Female	372 (10,341)	3.60% (3.24%-3.96%) [***]	3 (1-9) [ns]
	AA	139 (2,953)	4.71% (3.94%-5.47%)	4 (1-9)	Male	843 (31,327)	2.69% (2.51%-2.87%)	4 (1-11)
	Other	56 (2,231)	2.51% (1.86%-3.16%)	7 (2-17)				
	Unknown	1 (399)	NA	NA (NA-NA)				
Prostate	Caucasian	949 (233,486)	0.41% (0.38%-0.43%) [***]	21 (8-63) [ns]	Male	1,339 (309,918)	0.43% (0.41%-0.46%)	22 (8-63)
	AA	288 (49,941)	0.58% (0.51%-0.64%)	23 (9-65)				
	Other	96 (16,121)	0.60% (0.48%-0.71%)	19 (9-58)				
	Unknown	6 (10,370)	0.06% (0.01%-0.10%)	43 (26-NA)				
Testis	Caucasian	1,016 (14,094)	7.21% (6.78%-7.64%) [***]	NA (20-NA) [ns]	Male	1,129 (15,881)	7.11% (6.71%-7.51%)	NA (20-NA)
	AA	42 (471)	8.92% (6.34%-11.49%)	NA (NA-NA)				
	Other	68 (900)	7.56% (5.83%-9.28%)	NA (12-NA)				

	Unknown	3 (416)	NA	NA (NA-NA)				
Other GU	Caucasian	422 (7,226)	5.84% (5.30%-6.38%) *	5 (2-12) ^{ns}	Female	215 (2,736)	7.86% (6.85%-8.87%) ***	5 (2-11) ^{ns}
	AA	54 (696)	7.76% (5.77%-9.75%)	4 (1-9)	Male	314 (5,982)	5.25% (4.68%-5.81%)	6 (2-13)
	Other	52 (711)	7.31% (5.40%-9.23%)	4 (2-15)				
	Unknown	1 (85)	NA	10 (10-10)				
Ovary	Caucasian	1,346 (24,094)	5.59% (5.30%-5.88%) ***	16 (3-35) ***	Female	1,696 (29,789)	5.69% (5.43%-5.96%)	16 (2-36)
	AA	205 (2,604)	7.87% (6.84%-8.91%)	8 (2-28)				
	Other	141 (2,916)	4.84% (4.06%-5.61%)	19 (4-46)				
	Unknown	4 (175)	NA	NA (NA-NA)				
Uterus	Caucasian	905 (58,908)	1.54% (1.44%-1.64%) ***	8 (2-23) ^{ns}	Female	1,232 (73,342)	1.68% (1.59%-1.77%)	8 (2-22)
	AA	206 (6,850)	3.01% (2.60%-3.41%)	8 (2-16)				
	Other	117 (6,874)	1.70% (1.40%-2.01%)	9 (3-29)				
	Unknown	4 (710)	NA	30.5 (5-NA)				
Cervix	Caucasian	620 (15,415)	4.02% (3.71%-4.33%) ***	7 (2-18) ^{ns}	Female	844 (20,658)	4.09% (3.82%-4.36%)	7 (3-17)
	AA	136 (2,782)	4.89% (4.09%-5.69%)	6 (3-13)				
	Other	88 (2,226)	3.95% (3.14%-4.76%)	7 (4-25)				
	Unknown	0 (235)	NA	NA				
Other GYN	Caucasian	366 (8,733)	4.19% (3.77%-4.61%) *	5 (1-23) *	Female	457 (10,514)	4.35% (3.96%-4.74%)	5 (1-23)
	AA	52 (1,014)	5.13% (3.77%-6.49%)	2 (1-8)				
	Other	38 (646)	5.88% (4.07%-7.70%)	8 (3-43)				
	Unknown	1 (121)	NA	1 (1-1)				
Bone tumor	Caucasian	503 (4,155)	12.11% (11.11%-13.10%) ^{ns}	20 (8-NA) ^{ns}	Female	221 (2,205)	10.02% (8.77%-11.28%) ***	18 (8-NA) ^{ns}
	AA	57 (456)	12.50% (9.46%-15.54%)	13 (6-37)	Male	388 (2,874)	13.50% (12.25%-14.75%)	19 (8-59)
	Other	45 (429)	10.49% (7.59%-13.39%)	18 (9-37)				
	Unknown	4 (39)	NA	21 (14-NA)				
STS	Caucasian	1,574 (19,041)	8.27% (7.88%-8.66%) ***	8 (2-24) ^{ns}	Female	1,199 (12,286)	9.76% (9.23%-10.28%) ***	9 (2-23) ^{ns}
	AA	395 (3,328)	11.87% (10.77%-12.97%)	8 (3-21)	Male	982 (12,807)	7.67% (7.21%-8.13%)	8 (2-22)
	Other	203 (2,234)	9.09% (7.89%-10.28%)	9 (2-23)				
	Unknown	9 (490)	1.84% (0.65%-3.03%)	5 (3-NA)				
Skin melanoma	Caucasian	1,017 (99,591)	1.02% (0.96%-1.08%) ***	6 (2-20) ^{ns}	Female	302 (46,781)	0.65% (0.57%-0.72%) ***	6 (2-20) ^{ns}
	AA	18 (454)	3.96% (2.17%-5.76%)	6 (3-13)	Male	763 (60,506)	1.26% (1.17%-1.35%)	6 (2-20)
	Other	27 (910)	2.97% (1.86%-4.07%)	5 (3-12)				
	Unknown	3 (6,332)	NA	NA (NA-NA)				
Non-skin melanoma	Caucasian	93 (2,793)	3.33% (2.66%-4.00%) ^{ns}	6 (3-14) ^{ns}	Female	61 (1,689)	3.61% (2.72%-4.50%) ^{ns}	5 (3-14) ^{ns}
	AA	3 (97)	NA	3 (0-4)	Male	45 (1,395)	3.23% (2.30%-4.15%)	6 (3-14)
	Other	10 (154)	6.49% (2.60%-10.39%)	6 (5-14)				
	Unknown	0 (40)	NA	NA				
Skin cancer	Caucasian	30 (4,661)	0.64% (0.41%-0.87%) ^{ns}	9 (6-20) ^{ns}	Female	13 (2,253)	0.58% (0.26%-0.89%) ^{ns}	17 (8-34) ^{ns}
	AA	1 (207)	NA	17 (17-17)	Male	24 (3,092)	0.78% (0.47%-1.09%)	9 (5.5-18)
	Other	3 (242)	NA	8 (7-10)				
	Unknown	3 (235)	NA	33.5 (4-NA)				
Embryonal tumors	Caucasian	245 (3,229)	7.59% (6.67%-8.50%) ^{ns}	NA (19-NA) ^{ns}	Female	168 (1,999)	8.40% (7.19%-9.62%) ^{ns}	NA (NA-NA) ***
	AA	55 (575)	9.57% (7.16%-11.97%)	NA (NA-NA)	Male	166 (2,282)	7.27% (6.21%-8.34%)	NA (12-NA)
	Other	30 (419)	7.16% (4.69%-9.63%)	NA (23-NA)				
	Unknown	4 (58)	NA	NA (30-NA)				
All other	Caucasian	2,229 (24,926)	8.94% (8.59%-9.30%) *	4 (1-18) ***	Female	1,781 (20,228)	8.80% (8.41%-9.20%) ^{ns}	6 (1-24) ***
	AA	382 (4,696)	8.13% (7.35%-8.92%)	5 (1-24)	Male	1,122 (12,840)	8.74% (8.25%-9.23%)	4 (1-15)
	Other	274 (3,070)	8.93% (7.92%-9.93%)	8 (1-43)				
	Unknown	18 (376)	4.79% (2.63%-6.95%)	2 (0-15)				

[a] Prevalence was only calculated in category with more than 5 sLM cases.

[b] Survival data (in months) are shown as the median survival and interquartile range in cases with synchronous lung metastasis at diagnosis.

[c] Liver cancer here includes hepatocellular carcinoma and intrahepatic cholangiocarcinoma.

Abbreviations: sLM: synchronous lung metastasis; AA: African American; GI: gastrointestinal cancer; GU: genitourinary cancer; GYN: gynecologic cancer; STS: soft-tissue sarcoma; NA: non-applicable; ns: non-significant; *: p<0.05; ***: p<0.001% for intragroup survival comparison by Chi-square tests or log-rank tests.

Supplementary Table S2. Number of cases with synchronous lung metastasis and all cases, prevalence of synchronous lung metastasis and median survival with interquartile range by cancer type and age or county-level income.

Categories	Age	No. of sLM (All)	Prevalence [a]	Survival [b]	Income [c]	No. of sLM (All)	Prevalence [a]	Survival [b]
Brain	0 - 18	1 (2,577)	NA [ns]	NA (NA-NA) [ns]	1st	1 (1,120)	NA [ns]	18 (18-18) [ns]
	19 - 40	3 (4,280)	NA	16 (9-NA)	2nd	1 (2,363)	NA	7 (7-7)
	41 - 60	4 (9,379)	NA	15 (5-26)	3rd	1 (3,785)	NA	NA (NA-NA)
	61 - 80	8 (10,015)	0.08% (0.02%-0.14%)	2.5 (1.5-14.5)	4th	14 (20,193)	0.07% (0.03%-0.11%)	6.5 (2-23)
	80+	1 (1,234)	NA	4 (4-4)	Unk	0 (24)	NA	NA
Head and neck	0 - 18	4 (249)	NA [***]	34 (34-34) [***]	1st	118 (4,595)	2.57% (2.11%-3.03%) [***]	6 (2-12) [***]
	19 - 40	47 (3,089)	1.52% (1.09%-1.95%)	22 (11-32)	2nd	222 (8,884)	2.50% (2.17%-2.82%)	7 (3-14)
	41 - 60	598 (31,177)	1.92% (1.77%-2.07%)	9 (4-24)	3rd	235 (11,026)	2.13% (1.86%-2.40%)	7 (2-19)
	61 - 80	803 (34,246)	2.34% (2.18%-2.51%)	8 (3-18)	4th	995 (50,272)	1.98% (1.86%-2.10%)	10 (4-24)
	80+	122 (6,136)	1.99% (1.64%-2.34%)	5 (1-13)	Unk	4 (120)	NA	7 (4.5-15.5)
Thyroid	0 - 18	24 (1,308)	1.83% (1.11%-2.56%) [***]	NA (NA-NA) [***]	1st	34 (2,885)	1.18% (0.78%-1.57%) [ns]	12 (2-35) [ns]
	19 - 40	78 (22,756)	0.34% (0.27%-0.42%)	NA (NA-NA)	2nd	85 (6,207)	1.37% (1.08%-1.66%)	16 (3-79)
	41 - 60	242 (35,383)	0.68% (0.60%-0.77%)	14 (3-76)	3rd	124 (9,795)	1.27% (1.04%-1.49%)	11 (1-NA)
	61 - 80	522 (17,035)	3.06% (2.81%-3.32%)	9 (2-59)	4th	753 (59,019)	1.28% (1.19%-1.37%)	11 (2-83)
	80+	130 (1,521)	8.55% (7.14%-9.95%)	2 (0-14)	Unk	0 (97)	NA	NA
Breast	0 - 18	0 (8)	NA [***]	NA [***]	1st	323 (15,489)	2.09% (1.86%-2.31%) [***]	15 (4-37) [***]
	19 - 40	384 (22,835)	1.68% (1.51%-1.85%)	30 (13-58)	2nd	666 (31,952)	2.08% (1.93%-2.24%)	17 (4-41)
	41 - 60	2,228 (154,724)	1.44% (1.38%-1.50%)	24 (7-50)	3rd	710 (46,654)	1.52% (1.41%-1.63%)	20 (5-42)
	61 - 80	2,664 (152,615)	1.75% (1.68%-1.81%)	19 (4-48)	4th	4,260 (264,065)	1.61% (1.57%-1.66%)	21 (5-50)
	80+	696 (28,467)	2.44% (2.27%-2.62%)	8 (2-25)	Unk	13 (489)	2.66% (1.23%-4.08%)	15 (2-30)
Main bronchus	0 - 18	0 (12)	NA [***]	NA [***]	1st	138 (978)	14.11% (11.93%-16.29%) [***]	4 (1-12) [ns]
	19 - 40	14 (152)	9.21% (4.61%-13.81%)	8 (3-17)	2nd	246 (1,687)	14.58% (12.90%-16.27%)	3 (1-10)
	41 - 60	543 (3,311)	16.40% (15.14%-17.66%)	5 (1-13)	3rd	363 (2,026)	17.92% (16.25%-19.59%)	3 (1-9)
	61 - 80	1,106 (6,475)	17.08% (16.16%-18.00%)	3 (1-9)	4th	1,103 (6,176)	17.86% (16.90%-18.81%)	4 (1-10)
	80+	190 (928)	20.47% (17.88%-23.07%)	1 (0-6)	Unk	3 (11)	NA	2 (0-11)
Oesophagus	0 - 18	0 (2)	NA [*]	NA [***]	1st	116 (1,161)	9.99% (8.27%-11.72%) [ns]	4 (1-8) [*]
	19 - 40	35 (251)	13.94% (9.66%-18.23%)	6 (2-13)	2nd	215 (2,419)	8.89% (7.75%-10.02%)	4 (1-8)
	41 - 60	621 (6,005)	10.34% (9.57%-11.11%)	4 (2-10)	3rd	273 (2,967)	9.20% (8.16%-10.24%)	3 (1-8)
	61 - 80	1,117 (11,404)	9.79% (9.25%-10.34%)	4 (1-10)	4th	1,367 (13,474)	10.15% (9.64%-10.66%)	4 (1-10)
	80+	203 (2,406)	8.44% (7.33%-9.55%)	2 (1-5)	Unk	5 (47)	10.64% (1.82%-19.45%)	1 (1-7)
Stomach	0 - 18	4 (20)	NA [***]	6.5 (3-10) [***]	1st	81 (1,549)	5.23% (4.12%-6.34%) [ns]	3 (1-9) [ns]
	19 - 40	99 (1,426)	6.94% (5.62%-8.26%)	3 (1-7)	2nd	168 (2,947)	5.70% (4.86%-6.54%)	3 (1-9)
	41 - 60	564 (9,413)	5.99% (5.51%-6.47%)	4 (1-11)	3rd	196 (3,669)	5.34% (4.61%-6.07%)	3 (1-8)
	61 - 80	864 (15,714)	5.50% (5.14%-5.85%)	3 (1-9)	4th	1,299 (23,556)	5.51% (5.22%-5.81%)	3 (1-10)
	80+	225 (5,252)	4.28% (3.74%-4.83%)	2 (1-6)	Unk	12 (104)	11.54% (5.40%-17.68%)	3.5 (1-7.5)
Liver [d]	0 - 18	13 (78)	16.67% (8.40%-24.94%) [***]	7 (1-21) [***]	1st	105 (1,296)	8.10% (6.62%-9.59%) [ns]	2 (0-8) [ns]
	19 - 40	76 (549)	13.84% (10.95%-16.73%)	6 (1-14)	2nd	237 (2,759)	8.59% (7.54%-9.64%)	2 (0-6)
	41 - 60	856 (9,324)	9.18% (8.59%-9.77%)	2 (1-7)	3rd	303 (3,571)	8.49% (7.57%-9.40%)	2 (0-6)
	61 - 80	1,117 (14,052)	7.95% (7.50%-8.40%)	2 (0-5)	4th	1,594 (18,599)	8.57% (8.17%-8.97%)	2 (1-6)
	80+	183 (2,264)	8.08% (6.96%-9.21%)	2 (1-5)	Unk	6 (42)	14.29% (3.70%-24.87%)	1.5 (0-5)
Extrahepatic biliary tract	0 - 18	1 (5)	NA [***]	3 (3-3) [***]	1st	18 (607)	2.97% (1.62%-4.31%) [***]	4 (2-12) [ns]
	19 - 40	19 (270)	7.04% (3.99%-10.09%)	10 (3-18)	2nd	80 (1,283)	6.24% (4.91%-7.56%)	3 (1-8)
	41 - 60	229 (3,546)	6.46% (5.65%-7.27%)	4 (2-10)	3rd	115 (1,904)	6.04% (4.97%-7.11%)	3 (1-8)
	61 - 80	510 (7,873)	6.48% (5.93%-7.02%)	3 (1-8)	4th	660 (10,424)	6.33% (5.86%-6.80%)	3 (1-8)
	80+	117 (2,544)	4.60% (3.79%-5.41%)	2 (0-5)	Unk	3 (20)	NA	1 (0-9)
Pancreas	0 - 18	2 (53)	NA [***]	11 (3-19) [***]	1st	288 (2,569)	11.21% (9.99%-12.43%) [ns]	2 (1-5) [***]
	19 - 40	73 (992)	7.36% (5.73%-8.98%)	4 (2-10)	2nd	577 (5,213)	11.07% (10.22%-11.92%)	2 (1-6)
	41 - 60	1,418 (14,277)	9.93% (9.44%-10.42%)	3 (1-9)	3rd	736 (7,090)	10.38% (9.67%-11.09%)	3 (1-7)
	61 - 80	3,418 (30,171)	11.33% (10.97%-11.69%)	3 (1-8)	4th	4,070 (37,519)	10.85% (10.53%-11.16%)	3 (1-8)
	80+	771 (6,949)	11.10% (10.36%-11.83%)	2 (0-4)	Unk	11 (51)	21.57% (10.28%-32.86%)	2 (0-5)
Small intestine	0 - 18	0 (10)	NA [*]	NA [***]	1st	9 (569)	1.58% (0.56%-2.61%) [ns]	8 (2-13) [ns]
	19 - 40	6 (423)	1.42% (0.29%-2.55%)	12.5 (0-28.5)	2nd	30 (1,079)	2.78% (1.80%-3.76%)	7 (2-21)
	41 - 60	65 (3,397)	1.91% (1.45%-2.37%)	10 (3-37)	3rd	34 (1,441)	2.36% (1.58%-3.14%)	6 (1-17)
	61 - 80	134 (4,702)	2.85% (2.37%-3.33%)	8 (2-22)	4th	166 (6,535)	2.54% (2.16%-2.92%)	8 (2-22)
	80+	34 (1,096)	3.10% (2.08%-4.13%)	2 (1-6)	Unk	0 (4)	NA	NA
Colon & rectum	0 - 18	5 (63)	7.94% (1.26%-14.61%) [***]	14 (10-19) [***]	1st	582 (11,025)	5.28% (4.86%-5.70%) [*]	11 (3-24) [*]
	19 - 40	378 (7,048)	5.36% (4.84%-5.89%)	19 (9-33)	2nd	1,104 (20,099)	5.49% (5.18%-5.81%)	10 (2-24)
	41 - 60	3,563 (63,836)	5.58% (5.40%-5.76%)	16 (6-30)	3rd	1,300 (26,182)	4.97% (4.70%-5.23%)	11 (3-25)
	61 - 80	4,507 (86,417)	5.22% (5.07%-5.36%)	10 (2-24)	4th	6,629 (128,812)	5.15% (5.03%-5.27%)	12 (3-26)
	80+	1,192 (29,175)	4.09% (3.86%-4.31%)	3 (1-11)	Unk	30 (421)	7.13% (4.67%-9.58%)	7 (4-17)
Right colon	0 - 18	0 (19)	NA [***]	NA [***]	1st	194 (4,207)	4.61% (3.98%-5.25%) [***]	9 (2-21) [ns]
	19 - 40	57 (1,661)	3.43% (2.56%-4.31%)	15 (6-30)	2nd	327 (8,069)	4.05% (3.62%-4.48%)	8 (2-20)
	41 - 60	832 (17,008)	4.89% (4.57%-5.22%)	12 (3-23)	3rd	358 (10,493)	3.41% (3.06%-3.76%)	7 (2-20)
	61 - 80	1,433 (37,019)	3.87% (3.67%-4.07%)	9 (2-21)	4th	1,890 (49,468)	3.82% (3.65%-3.99%)	9 (2-21)
	80+	455 (16,676)	2.73% (2.48%-2.98%)	3 (1-10)	Unk	8 (146)	5.48% (1.79%-9.17%)	13 (5.5-24)
Left colon	0 - 18	1 (22)	NA [*]	19 (19-19) [***]	1st	193 (3,846)	5.02% (4.33%-5.71%) [ns]	12 (4-24) [***]
	19 - 40	167 (2,791)	5.98% (5.10%-6.86%)	20 (12-37)	2nd	400 (6,667)	6.00% (5.43%-6.57%)	13 (3-25)
	41 - 60	1,357 (25,121)	5.40% (5.12%-5.68%)	18 (6-33)	3rd	455 (8,703)	5.23% (4.76%-5.70%)	13 (3-26)
	61 - 80	1,512 (28,653)	5.28% (5.02%-5.54%)	11 (3-25)	4th	2,311 (44,629)	5.18% (4.97%-5.38%)	14 (4-29)
	80+	333 (7,427)	4.48% (4.01%-4.95%)	3 (1-11)	Unk	11 (169)	6.51% (2.79%-10.23%)	5 (2-11)
Unspecified colon	0 - 18	1 (4)	NA [ns]	0 (0-0) [***]	1st	44 (309)	14.24% (10.34%-18.14%) [ns]	5 (2-12) [*]
	19 - 40	24 (160)	15.00% (9.47%-20.53%)	6 (1-18)	2nd	83 (515)	16.12% (12.94%-19.29%)	2 (0-9)
	41 - 60	210 (1,290)	16.28% (14.26%-18.29%)	6 (1-17)	3rd	106 (660)	16.06% (13.26%-18.86%)	4 (1-19)
	61 - 80	367 (2,287)	16.05% (14.54%-17.55%)	3 (1-12)	4th	513 (3,239)	15.84% (14.58%-17.10%)	3 (1-13)

	80+	144 (986)	14.60% (12.40%-16.81%)	1 (0-6)	Unk	0 (4)	NA	NA
Rectum	0 - 18	3 (18)	NA ***	14 (10-40) ***	1st	151 (2,663)	5.67% (4.79%-6.55%) ns	17 (7-30) ns
	19 - 40	130 (2,436)	5.34% (4.44%-6.23%)	24 (10-37)	2nd	294 (4,848)	6.06% (5.39%-6.74%)	13 (4-27)
	41 - 60	1,164 (20,417)	5.70% (5.38%-6.02%)	19 (8-34)	3rd	381 (6,326)	6.02% (5.44%-6.61%)	15 (4-28)
	61 - 80	1,195 (18,458)	6.47% (6.12%-6.83%)	13 (4-27)	4th	1,915 (31,476)	6.08% (5.82%-6.35%)	16 (6-30)
	80+	260 (4,086)	6.36% (5.61%-7.11%)	6 (2-18)	Unk	11 (102)	10.78% (4.76%-16.80%)	11 (6-17)
Anus	0 - 18	NA ()	#VALUE!	NA	1st	9 (469)	1.92% (0.68%-3.16%) ns	10 (6-12) ns
	19 - 40	7 (313)	2.24% (0.60%-3.87%)	11.5 (6-21)	2nd	20 (977)	2.05% (1.16%-2.94%)	13 (5-23)
	41 - 60	76 (4,446)	1.71% (1.33%-2.09%)	15 (6-46)	3rd	24 (1,327)	1.81% (1.09%-2.53%)	11 (6-36)
	61 - 80	81 (3,864)	2.10% (1.64%-2.55%)	10 (5-17)	4th	129 (6,623)	1.95% (1.61%-2.28%)	11 (5-22)
	80+	19 (782)	2.43% (1.35%-3.51%)	6 (3-9)	Unk	1 (9)	NA	6 (6-6)
Other GI	0 - 18	1 (323)	NA ***	2 (2-2) ***	1st	17 (364)	4.67% (2.50%-6.84%) ns	0 (0-2) ns
	19 - 40	21 (1,350)	1.56% (0.90%-2.22%)	8 (2-18)	2nd	65 (791)	8.22% (6.30%-10.13%)	2 (0-8)
	41 - 60	171 (3,145)	5.44% (4.64%-6.23%)	3 (1-9)	3rd	99 (1,132)	8.75% (7.10%-10.39%)	2 (1-6)
	61 - 80	376 (3,117)	12.06% (10.92%-13.21%)	2 (0-7)	4th	496 (6,309)	7.86% (7.20%-8.53%)	2 (0-7)
	80+	111 (676)	16.42% (13.63%-19.21%)	1 (0-2)	Unk	3 (15)	NA	0 (0-2)
Kidney	0 - 18	7 (76)	9.21% (2.71%-15.71%) ***	7 (3-9) ***	1st	330 (3,892)	8.48% (7.60%-9.35%) ns	6 (2-14) ***
	19 - 40	168 (4,390)	3.83% (3.26%-4.39%)	9 (4-20)	2nd	671 (7,738)	8.67% (8.04%-9.30%)	7 (2-20)
	41 - 60	2,316 (28,812)	8.04% (7.72%-8.35%)	9 (3-26)	3rd	922 (10,582)	8.71% (8.18%-9.25%)	8 (2-21)
	61 - 80	2,936 (32,761)	8.96% (8.65%-9.27%)	7 (2-22)	4th	3,890 (47,276)	8.23% (7.98%-8.48%)	8 (3-23)
	80+	396 (3,566)	11.10% (10.07%-12.14%)	3 (1-9)	Unk	10 (117)	8.55% (3.48%-13.61%)	5.5 (1-12.5)
Bladder	0 - 18	1 (3)	NA ***	28 (28-28) ***	1st	70 (1,883)	3.72% (2.86%-4.57%) *	3 (1-12) ns
	19 - 40	16 (335)	4.78% (2.49%-7.06%)	6 (3.5-18)	2nd	147 (4,184)	3.51% (2.96%-4.07%)	5 (2-10)
	41 - 60	251 (7,506)	3.34% (2.94%-3.75%)	6 (2-13)	3rd	168 (5,685)	2.96% (2.51%-3.40%)	4 (1-11)
	61 - 80	676 (23,734)	2.85% (2.64%-3.06%)	4 (1-11)	4th	829 (29,886)	2.77% (2.59%-2.96%)	4 (1-11)
	80+	271 (10,090)	2.69% (2.37%-3.00%)	3 (1-7)	Unk	1 (30)	NA	1 (1-1)
Prostate	0 - 18	0 (0)		NA	1st	58 (15,969)	0.36% (0.27%-0.46%) ns	24 (8-77) ns
	19 - 40	1 (304)	NA	NA (NA-NA)	2nd	147 (32,744)	0.45% (0.38%-0.52%)	23 (7-NA)
	41 - 60	276 (87,961)	0.31% (0.28%-0.35%)	31 (11-NA)	3rd	164 (40,214)	0.41% (0.35%-0.47%)	19 (6-42)
	61 - 80	811 (204,869)	0.40% (0.37%-0.42%)	25 (9-67)	4th	969 (220,818)	0.44% (0.41%-0.47%)	22 (9-62)
	80+	251 (16,784)	1.50% (1.31%-1.68%)	11 (3-28)	Unk	1 (173)	NA	8 (8-8)
Testis	0 - 18	77 (650)	11.85% (9.36%-14.33%) ***	NA (NA-NA) ***	1st	40 (546)	7.33% (5.14%-9.51%) ns	NA (36-NA) ns
	19 - 40	850 (11,072)	7.68% (7.18%-8.17%)	NA (20-NA)	2nd	95 (1,159)	8.20% (6.62%-9.78%)	NA (23-NA)
	41 - 60	176 (3,710)	4.74% (4.06%-5.43%)	NA (11-NA)	3rd	162 (2,044)	7.93% (6.75%-9.10%)	NA (26-NA)
	61 - 80	25 (426)	5.87% (3.64%-8.10%)	12 (2-NA)	4th	831 (12,100)	6.87% (6.42%-7.32%)	NA (19-NA)
	80+	1 (23)	NA	NA (NA-NA)	Unk	1 (32)	NA	NA (NA-NA)
Other GU	0 - 18	0 (2)	NA ns	NA ***	1st	20 (475)	4.21% (2.40%-6.02%) ns	5 (1-7) ns
	19 - 40	8 (146)	5.48% (1.79%-9.17%)	5.5 (5-10)	2nd	50 (853)	5.86% (4.29%-7.44%)	4 (2-9)
	41 - 60	98 (1,784)	5.49% (4.44%-6.55%)	6 (2-14)	3rd	78 (1,262)	6.18% (4.85%-7.51%)	5 (2-11)
	61 - 80	315 (4,892)	6.44% (5.75%-7.13%)	6 (2-14)	4th	379 (6,119)	6.19% (5.59%-6.80%)	5 (2-13)
	80+	108 (1,894)	5.70% (4.66%-6.75%)	2 (1-6)	Unk	2 (9)	NA	5.5 (4-7)
Ovary	0 - 18	7 (446)	1.57% (0.42%-2.72%) ***	48 (34-NA) ***	1st	73 (1,190)	6.13% (4.77%-7.50%) ns	8 (2-25) ***
	19 - 40	60 (2,520)	2.38% (1.79%-2.98%)	12 (4-39)	2nd	142 (2,517)	5.64% (4.74%-6.54%)	5 (1-24)
	41 - 60	493 (11,865)	4.16% (3.80%-4.51%)	23 (5-50)	3rd	213 (3,669)	5.81% (5.05%-6.56%)	12 (3-31)
	61 - 80	902 (12,264)	7.35% (6.89%-7.82%)	17 (2-34)	4th	1,268 (22,390)	5.66% (5.36%-5.97%)	18 (3-38)
	80+	234 (2,694)	8.69% (7.62%-9.75%)	3 (1-13)	Unk	0 (23)	NA	NA
Uterus	0 - 18	0 (7)	NA ***	NA *	1st	75 (3,093)	2.42% (1.88%-2.97%) ***	8 (1-21) ns
	19 - 40	35 (3,192)	1.10% (0.74%-1.46%)	8 (1-NA)	2nd	128 (6,408)	2.00% (1.65%-2.34%)	8 (2-20)
	41 - 60	421 (30,250)	1.39% (1.26%-1.52%)	7 (2-24)	3rd	168 (9,065)	1.85% (1.58%-2.13%)	8 (1-24)
	61 - 80	677 (35,825)	1.89% (1.75%-2.03%)	9 (2-23)	4th	860 (54,721)	1.57% (1.47%-1.68%)	8 (2-22)
	80+	99 (4,068)	2.43% (1.96%-2.91%)	6 (2-14)	Unk	1 (55)	NA	NA (NA-NA)
Cervix	0 - 18	1 (9)	NA ***	5 (5-5) ***	1st	57 (1,184)	4.81% (3.59%-6.03%) ns	7 (4-24) ns
	19 - 40	98 (6,125)	1.60% (1.29%-1.91%)	10 (4-20)	2nd	94 (2,235)	4.21% (3.37%-5.04%)	8 (3-18)
	41 - 60	393 (9,634)	4.08% (3.68%-4.47%)	7 (3-18)	3rd	113 (2,857)	3.96% (3.24%-4.67%)	6 (2-15)
	61 - 80	302 (4,203)	7.19% (6.40%-7.97%)	7 (2-16)	4th	578 (14,353)	4.03% (3.71%-4.35%)	7 (3-17)
	80+	50 (687)	7.28% (5.34%-9.22%)	4 (2-9)	Unk	2 (29)	NA	NA (NA-NA)
Other GYN	0 - 18	0 (3)	NA ns	NA ***	1st	20 (623)	3.21% (1.83%-4.59%) ns	7 (1-NA) *
	19 - 40	12 (398)	3.02% (1.34%-4.70%)	20 (5-NA)	2nd	56 (1,120)	5.00% (3.72%-6.28%)	5 (1-20)
	41 - 60	148 (3,405)	4.35% (3.66%-5.03%)	6 (1-24)	3rd	66 (1,587)	4.16% (3.18%-5.14%)	2 (0-14)
	61 - 80	224 (4,921)	4.55% (3.97%-5.13%)	5 (1-23)	4th	314 (7,167)	4.38% (3.91%-4.86%)	6 (1-24)
	80+	73 (1,787)	4.09% (3.17%-5.00%)	3 (0-10)	Unk	1 (17)	NA	5 (5-5)
Bone tumor	0 - 18	264 (1,489)	17.73% (15.79%-19.67%) ***	35 (17-NA) ***	1st	25 (184)	13.59% (8.64%-18.54%) ns	17 (9-NA) ns
	19 - 40	131 (1,286)	10.19% (8.53%-11.84%)	18 (10-47)	2nd	56 (457)	12.25% (9.25%-15.26%)	18 (7-46)
	41 - 60	100 (1,248)	8.01% (6.51%-9.52%)	8 (3-18)	3rd	82 (672)	12.20% (9.73%-14.68%)	20 (7-47)
	61 - 80	93 (868)	10.71% (8.66%-12.77%)	9 (3-23)	4th	445 (3,756)	11.85% (10.81%-12.88%)	19 (8-NA)
	80+	21 (188)	11.17% (6.67%-15.67%)	4 (1-7)	Unk	1 (10)	NA	9 (9-9)
STS	0 - 18	149 (1,533)	9.72% (8.24%-11.20%) ***	23 (10-NA) ***	1st	104 (1,049)	9.91% (8.11%-11.72%) *	12 (2-22) ns
	19 - 40	323 (4,083)	7.91% (7.08%-8.74%)	15 (5-32)	2nd	223 (2,204)	10.12% (8.86%-11.38%)	7 (2-21)
	41 - 60	804 (8,794)	9.14% (8.54%-9.74%)	10 (3-24)	3rd	274 (3,258)	8.41% (7.46%-9.36%)	9 (3-22)
	61 - 80	755 (8,387)	9.00% (8.39%-9.61%)	5 (1-17)	4th	1,580 (18,557)	8.51% (8.11%-8.92%)	8 (2-23)
	80+	150 (2,296)	6.53% (5.52%-7.54%)	2 (1-7)	Unk	0 (25)	NA	NA
Skin melanoma	0 - 18	1 (463)	NA ***	0 (0-0) ***	1st	37 (3,516)	1.05% (0.72%-1.39%) ns	7 (4-27) *
	19 - 40	70 (12,839)	0.55% (0.42%-0.67%)	10 (4-22)	2nd	84 (8,156)	1.03% (0.81%-1.25%)	5 (1-12)
	41 - 60	337 (38,800)	0.87% (0.78%-0.96%)	9 (3-23)	3rd	150 (14,272)	1.05% (0.88%-1.22%)	5 (2-11)
	61 - 80	499 (43,874)	1.14% (1.04%-1.24%)	5 (2-19)	4th	794 (81,305)	0.98% (0.91%-1.04%)	7 (2-22)
	80+	158 (11,311)	1.40% (1.18%-1.61%)	6 (2-13)	Unk	0 (38)	NA	NA
Non-skin melanoma	0 - 18	0 (14)	NA ns	NA *	1st	4 (149)	NA ns	10 (4-38) ns
	19 - 40	6 (236)	2.54% (0.53%-4.55%)	13 (7-27)	2nd	9 (281)	3.20% (1.14%-5.26%)	10 (1-18)
	41 - 60	27 (1,004)	2.69% (1.69%-3.69%)	7 (5-NA)	3rd	13 (463)	2.81% (1.30%-4.31%)	5 (3-6)
	61 - 80	56 (1,434)	3.91% (2.90%-4.91%)	6 (3-14)	4th	80 (2,187)	3.66% (2.87%-4.44%)	6 (3-14)
	80+	17 (396)	4.29% (2.30%-6.29%)	4 (2-6)	Unk	0 (4)	NA	NA

Skin cancer	0 - 18	0 (15)	NA [ns]	NA [ns]	1st	2 (203)	NA [ns]	16.5 (6-NA) [ns]
	19 - 40	0 (158)	NA	NA	2nd	5 (504)	0.99% (0.13%-1.86%)	10 (8-11)
	41 - 60	12 (1,013)	1.18% (0.52%-1.85%)	17 (6.5-48.5)	3rd	1 (700)	NA	25 (25-25)
	61 - 80	16 (2,683)	0.60% (0.31%-0.89%)	9 (5-11)	4th	29 (3,934)	0.74% (0.47%-1.00%)	8 (5-18)
	80+	9 (1,476)	0.61% (0.21%-1.01%)	10 (6-21)	Unk	0 (4)	NA	NA
Embryonal tumors	0 - 18	312 (3,524)	8.85% (7.92%-9.79%) [***]	NA (33-NA) [***]	1st	11 (157)	7.01% (3.01%-11.00%) [ns]	16 (4-57) [*]
	19 - 40	8 (311)	2.57% (0.81%-4.33%)	50 (6-NA)	2nd	30 (396)	7.58% (4.97%-10.18%)	NA (12-NA)
	41 - 60	5 (260)	1.92% (0.25%-3.59%)	16 (3-16)	3rd	49 (575)	8.52% (6.24%-10.80%)	NA (16-NA)
	61 - 80	9 (170)	5.29% (1.93%-8.66%)	4 (1-12)	4th	243 (3,140)	7.74% (6.80%-8.67%)	NA (26-NA)
	80+	0 (16)	NA	NA	Unk	1 (13)	NA	NA (NA-NA)
All other	0 - 18	52 (997)	5.22% (3.84%-6.60%) [***]	46 (16-NA) [***]	1st	135 (1,566)	8.62% (7.23%-10.01%) [ns]	2 (0-10) [***]
	19 - 40	285 (2,497)	11.41% (10.17%-12.66%)	24 (6-NA)	2nd	264 (3,194)	8.27% (7.31%-9.22%)	4 (1-13)
	41 - 60	828 (9,649)	8.58% (8.02%-9.14%)	7 (1-31)	3rd	368 (4,271)	8.62% (7.77%-9.46%)	5 (1-20)
	61 - 80	1,340 (15,709)	8.53% (8.09%-8.97%)	4 (1-13)	4th	2,130 (24,001)	8.87% (8.51%-9.23%)	5 (1-21)
	80+	398 (4,216)	9.44% (8.56%-10.32%)	2 (0-8)	Unk	6 (36)	16.67% (4.49%-28.84%)	0.5 (0-13)

[a] Prevalence was only calculated in category with more than 5 sLM cases.

[b] Survival data (in months) are shown as the median survival and interquartile range in cases with synchronous lung metastasis at diagnosis.

[c] The county-level income is presented as quantiles here.

[d] Liver cancer here includes hepatocellular carcinoma and intrahepatic cholangiocarcinoma.

Abbreviations: sLM: synchronous lung metastasis; AA: African American; Unk: unknown; GI: gastrointestinal cancer; GU: genitourinary cancer; GYN: gynecologic cancer; STS: soft-tissue sarcoma; NA: non-applicable; ns: non-significant; *: p<0.05; ***: p<0.001% for intragroup survival comparison by Chi-square tests or log-rank tests.

Supplementary Table S3. Number of cases with synchronous lung metastasis and all cases, prevalence of synchronous lung metastasis and median survival with interquartile range by cancer type and county-level education or county-level residence type.

Categories	Education [a]	No. of sLM (All)	Prevalence [b]	Survival [c]	Residence	No. of sLM (All)	Prevalence [b]	Survival [c]
Brain	1st	1 (898)	NA ^{ns}	18 (18-18) ^{ns}	Metro	16 (24,543)	0.07% (0.03%-0.10%) ^{ns}	8 (2.5-23) ^{ns}
	2nd	1 (2,082)	NA	NA (NA-NA)	Urban	1 (2,586)	NA	18 (18-18)
	3rd	2 (4,899)	NA	4.5 (2-7)	Rural	0 (355)	NA	NA
	4th	13 (19,582)	0.07% (0.03%-0.10%)	9 (3-23)	Unknown	0 (1)	NA	NA
	Unk	0 (24)	NA	NA				
Head and neck	1st	88 (3,637)	2.42% (1.92%-2.92%) ^{ns}	7 (3-14) *	Metro	1,357 (64,650)	2.10% (1.99%-2.21%) ^{ns}	9 (3-22) ***
	2nd	148 (6,662)	2.22% (1.87%-2.58%)	6 (2-19)	Urban	181 (8,868)	2.04% (1.75%-2.34%)	6 (2-13)
	3rd	327 (14,546)	2.25% (2.01%-2.49%)	8 (3-18)	Rural	36 (1,376)	2.62% (1.77%-3.46%)	8 (2-15)
	4th	1,007 (49,932)	2.02% (1.89%-2.14%)	9 (4-23)	Unknown	0 (3)	NA	NA
	Unk	4 (120)	NA	7 (4.5-15.5)				
Thyroid	1st	31 (2,175)	1.43% (0.93%-1.92%) *	7 (1-35) ^{ns}	Metro	916 (71,055)	1.29% (1.21%-1.37%) ^{ns}	11 (2-83) ^{ns}
	2nd	93 (5,316)	1.75% (1.40%-2.10%)	11 (2-NA)	Urban	71 (6,062)	1.17% (0.90%-1.44%)	16 (1-79)
	3rd	161 (13,052)	1.23% (1.04%-1.42%)	8 (1-65)	Rural	9 (878)	1.03% (0.36%-1.69%)	5 (1-22)
	4th	711 (57,363)	1.24% (1.15%-1.33%)	12 (2-79)	Unknown	0 (8)	NA	NA
	Unk	0 (97)	NA	NA				
Breast	1st	196 (10,683)	1.83% (1.58%-2.09%) ***	16 (4-49) *	Metro	5,385 (323,451)	1.66% (1.62%-1.71%) ^{ns}	20 (5-47) ^{ns}
	2nd	436 (24,455)	1.78% (1.62%-1.95%)	20 (5-40)	Urban	505 (30,732)	1.64% (1.50%-1.79%)	20 (5-47)
	3rd	1,078 (60,599)	1.78% (1.67%-1.88%)	18 (4-41)	Rural	82 (4,398)	1.86% (1.46%-2.26%)	18 (4-44)
	4th	4,249 (262,423)	1.62% (1.57%-1.67%)	21 (5-49)	Unknown	0 (68)	NA	NA
	Unk	13 (489)	2.66% (1.23%-4.08%)	15 (2-30)				
Main bronchus	1st	130 (927)	14.02% (11.79%-16.26%) ***	3 (1-10) ^{ns}	Metro	1,554 (8,947)	17.37% (16.58%-18.15%) ^{ns}	4 (1-11) ^{ns}
	2nd	199 (1,291)	15.41% (13.44%-17.38%)	3 (1-9)	Urban	248 (1,638)	15.14% (13.40%-16.88%)	3 (1-9)
	3rd	382 (2,418)	15.80% (14.34%-17.25%)	4 (1-10)	Rural	51 (293)	17.41% (13.06%-21.75%)	4 (1-11)
	4th	1,139 (6,231)	18.28% (17.32%-19.24%)	4 (1-11)	Unknown	0 (0)		NA
	Unk	3 (11)	NA	2 (0-11)				
Oesophagus	1st	80 (869)	9.21% (7.28%-11.13%) ^{ns}	4 (1-8) ^{ns}	Metro	1,727 (17,303)	9.98% (9.53%-10.43%) ^{ns}	4 (1-10) *
	2nd	148 (1,785)	8.29% (7.01%-9.57%)	4 (1-8)	Urban	220 (2,396)	9.18% (8.03%-10.34%)	3 (1-8)
	3rd	412 (4,077)	10.11% (9.18%-11.03%)	3 (1-9)	Rural	29 (365)	7.95% (5.17%-10.72%)	4 (1-8)
	4th	1,331 (13,290)	10.02% (9.50%-10.53%)	4 (1-10)	Unknown	0 (4)	NA	NA
	Unk	5 (47)	10.64% (1.82%-19.45%)	1 (1-7)				
Stomach	1st	53 (1,066)	4.97% (3.67%-6.28%) *	2 (1-5) ^{ns}	Metro	1,587 (28,792)	5.51% (5.25%-5.78%) ^{ns}	3 (1-9) ^{ns}
	2nd	116 (2,275)	5.10% (4.19%-6.00%)	3 (1-11)	Urban	140 (2,573)	5.44% (4.56%-6.32%)	3 (1-7)
	3rd	274 (5,373)	5.10% (4.51%-5.69%)	4 (1-9)	Rural	29 (459)	6.32% (4.09%-8.54%)	3 (1-6)
	4th	1,301 (23,007)	5.65% (5.36%-5.95%)	3 (1-9)	Unknown	0 (1)	NA	NA
	Unk	12 (104)	11.54% (5.40%-17.68%)	3.5 (1-7.5)				
Liver [d]	1st	78 (871)	8.96% (7.06%-10.85%) ^{ns}	2 (0-7) ^{ns}	Metro	2,017 (23,732)	8.50% (8.14%-8.85%) ^{ns}	2 (1-6) ^{ns}
	2nd	183 (2,076)	8.82% (7.60%-10.03%)	2 (0-6)	Urban	202 (2,227)	9.07% (7.88%-10.26%)	1 (0-5)
	3rd	418 (4,814)	8.68% (7.89%-9.48%)	2 (0-5)	Rural	26 (306)	8.50% (5.37%-11.62%)	3.5 (1-11)
	4th	1,560 (18,464)	8.45% (8.05%-8.85%)	2 (1-6)	Unknown	0 (2)	NA	NA
	Unk	6 (42)	14.29% (3.70%-24.87%)	1.5 (0-5)				
Extrahepatic biliary tract	1st	21 (441)	4.76% (2.77%-6.75%) ^{ns}	2.5 (2-5) ^{ns}	Metro	807 (12,834)	6.29% (5.87%-6.71%) ^{ns}	3 (1-8) ^{ns}
	2nd	56 (1,077)	5.20% (3.87%-6.53%)	4 (1-10)	Urban	61 (1,214)	5.02% (3.80%-6.25%)	4 (1-7)
	3rd	136 (2,443)	5.57% (4.66%-6.48%)	3 (1-9)	Rural	8 (190)	4.21% (1.35%-7.07%)	2.5 (0-7)
	4th	660 (10,257)	6.43% (5.96%-6.91%)	3 (1-8)	Unknown	0 (0)		NA
	Unk	3 (20)	NA	1 (0-9)				
Pancreas	1st	173 (1,744)	9.92% (8.52%-11.32%) *	2 (1-6) ***	Metro	5,093 (46,910)	10.86% (10.58%-11.14%) ^{ns}	3 (1-8) ^{ns}
	2nd	470 (4,034)	11.65% (10.66%-12.64%)	2 (1-6)	Urban	506 (4,788)	10.57% (9.70%-11.44%)	2 (1-7)
	3rd	1,051 (9,572)	10.98% (10.35%-11.61%)	2 (1-7)	Rural	83 (742)	11.19% (8.92%-13.45%)	3 (1-6)
	4th	3,977 (37,041)	10.74% (10.42%-11.05%)	3 (1-8)	Unknown	0 (2)	NA	NA
	Unk	11 (51)	21.57% (10.28%-32.86%)	2 (0-5)				
Small intestine	1st	8 (380)	2.11% (0.66%-3.55%) ^{ns}	8 (1-NA) ^{ns}	Metro	218 (8,471)	2.57% (2.24%-2.91%) ^{ns}	8 (2-21) ^{ns}
	2nd	18 (796)	2.26% (1.23%-3.29%)	11 (6-44)	Urban	20 (1,038)	1.93% (1.09%-2.76%)	8 (1.5-21)
	3rd	34 (1,806)	1.88% (1.26%-2.51%)	4 (1-11)	Rural	1 (119)	NA	NA (NA-NA)
	4th	179 (6,642)	2.69% (2.31%-3.08%)	8 (2-26)	Unknown	0 (0)		NA
	Unk	0 (4)	NA	NA				
Colon & rectum	1st	434 (8,354)	5.20% (4.72%-5.67%) *	10 (3-23) *	Metro	8,436 (162,938)	5.18% (5.07%-5.29%) ^{ns}	11 (3-25) ^{ns}
	2nd	765 (15,873)	4.82% (4.49%-5.15%)	10 (2-25)	Urban	1,013 (20,201)	5.01% (4.71%-5.32%)	11 (3-25)
	3rd	1,891 (35,129)	5.38% (5.15%-5.62%)	11 (3-25)	Rural	196 (3,390)	5.78% (5.00%-6.57%)	11 (4-21)
	4th	6,525 (126,762)	5.15% (5.03%-5.27%)	12 (3-25)	Unknown	0 (10)	NA	NA
	Unk	30 (421)	7.13% (4.67%-9.58%)	7 (4-17)				
Right colon	1st	136 (3,167)	4.29% (3.59%-5.00%) ^{ns}	6 (2-19) ^{ns}	Metro	2,410 (62,941)	3.83% (3.68%-3.98%) ^{ns}	8 (2-20) ^{ns}
	2nd	230 (6,144)	3.74% (3.27%-4.22%)	7 (2-20)	Urban	302 (8,104)	3.73% (3.31%-4.14%)	8 (2-21)
	3rd	531 (13,916)	3.82% (3.50%-4.13%)	8 (2-19)	Rural	65 (1,336)	4.87% (3.71%-6.02%)	8 (3-23)
	4th	1,872 (49,010)	3.82% (3.65%-3.99%)	9 (2-21)	Unknown	0 (2)	NA	NA
	Unk	8 (146)	5.48% (1.79%-9.17%)	13 (5.5-24)				
Left colon	1st	150 (2,924)	5.13% (4.33%-5.93%) ^{ns}	12 (4-23) ***	Metro	2,953 (56,143)	5.26% (5.08%-5.44%) ^{ns}	13 (3-29) ^{ns}
	2nd	264 (5,394)	4.89% (4.32%-5.47%)	12 (3-26)	Urban	350 (6,727)	5.20% (4.67%-5.73%)	14 (4-26)
	3rd	656 (11,727)	5.59% (5.18%-6.01%)	13 (3-27)	Rural	67 (1,139)	5.88% (4.52%-7.25%)	14 (4-21)
	4th	2,289 (43,800)	5.23% (5.02%-5.43%)	14 (4-29)	Unknown	0 (5)	NA	NA
	Unk	11 (169)	6.51% (2.79%-10.23%)	5 (2-11)				
Unspecified colon	1st	28 (221)	12.67% (8.28%-17.06%) ^{ns}	3 (0.5-11) ^{ns}	Metro	667 (4,152)	16.06% (14.95%-17.18%) ^{ns}	3 (1-12) ^{ns}
	2nd	53 (377)	14.06% (10.55%-17.57%)	2 (1-9)	Urban	69 (499)	13.83% (10.80%-16.86%)	3 (1-14)
	3rd	158 (925)	17.08% (14.66%-19.51%)	4 (1-11)	Rural	10 (75)	13.33% (5.64%-21.03%)	8 (2-13)

	4th	507 (3,200)	15.84% (14.58%-17.11%)	4 (1-13)	Unknown	0 (1)	NA	NA
	Unk	0 (4)	NA	NA				
Rectum	1st	120 (2,042)	5.88% (4.86%-6.90%) ^{ns}	12 (5-29) ^{ns}	Metro	2,406 (39,702)	6.06% (5.83%-6.29%) ^{ns}	16 (5-30) *
	2nd	218 (3,958)	5.51% (4.80%-6.22%)	16 (5-29)	Urban	292 (4,871)	5.99% (5.33%-6.66%)	13 (6-27)
	3rd	546 (8,561)	6.38% (5.86%-6.90%)	15 (4-30)	Rural	54 (840)	6.43% (4.77%-8.09%)	11 (5-21)
	4th	1,857 (30,752)	6.04% (5.77%-6.30%)	16 (6-29)	Unknown	0 (2)	NA	NA
	Unk	11 (102)	10.78% (4.76%-16.80%)	11 (6-17)				
Anus	1st	7 (332)	2.11% (0.56%-3.65%) *	16 (10-16) ^{ns}	Metro	161 (8,318)	1.94% (1.64%-2.23%) ^{ns}	12 (5-23) ^{ns}
	2nd	18 (771)	2.33% (1.27%-3.40%)	8 (5-35)	Urban	18 (946)	1.90% (1.03%-2.77%)	7 (4-13)
	3rd	20 (1,695)	1.18% (0.67%-1.69%)	12 (6-16)	Rural	4 (139)	NA	13 (8-16)
	4th	137 (6,598)	2.08% (1.73%-2.42%)	11 (5-23)	Unknown	0 (2)	NA	NA
	Unk	1 (9)	NA	6 (6-6)				
Other GI	1st	14 (272)	5.15% (2.52%-7.77%) ^{ns}	1 (0-2) ^{ns}	Metro	633 (7,727)	8.19% (7.58%-8.80%) ***	2 (0-7) ^{ns}
	2nd	52 (593)	8.77% (6.49%-11.05%)	1 (0-3)	Urban	40 (782)	5.12% (3.57%-6.66%)	2 (1-4)
	3rd	124 (1,535)	8.08% (6.71%-9.44%)	1 (0-7)	Rural	7 (102)	6.86% (1.96%-11.77%)	2 (0-8)
	4th	487 (6,196)	7.86% (7.19%-8.53%)	2 (0-7)	Unknown	0 (0)		NA
	Unk	3 (15)	NA	0 (0-2)				
Kidney	1st	254 (3,073)	8.27% (7.29%-9.24%) *	7 (3-17) ***	Metro	5,023 (61,101)	8.22% (8.00%-8.44%) ***	8 (2-23) ***
	2nd	602 (6,399)	9.41% (8.69%-10.12%)	7 (2-18)	Urban	700 (7,370)	9.50% (8.83%-10.17%)	7 (2-17)
	3rd	1,146 (13,579)	8.44% (7.97%-8.91%)	7 (2-19)	Rural	100 (1,132)	8.83% (7.18%-10.49%)	5 (2-14)
	4th	3,811 (46,437)	8.21% (7.96%-8.46%)	8 (3-24)	Unknown	0 (2)	NA	NA
	Unk	10 (117)	8.55% (3.48%-13.61%)	5.5 (1-12.5)				
Bladder	1st	66 (1,492)	4.42% (3.38%-5.47%) ***	3 (1-9) ^{ns}	Metro	1,064 (36,649)	2.90% (2.73%-3.08%) *	4 (1-11) ^{ns}
	2nd	103 (3,289)	3.13% (2.54%-3.73%)	4 (2-9)	Urban	120 (4,385)	2.74% (2.25%-3.22%)	4 (1-8)
	3rd	226 (7,795)	2.90% (2.53%-3.27%)	4 (2-11)	Rural	31 (633)	4.90% (3.22%-6.58%)	4 (2-12)
	4th	819 (29,062)	2.82% (2.63%-3.01%)	4 (1-11)	Unknown	0 (1)	NA	NA
	Unk	1 (30)	NA	1 (1-1)				
Prostate	1st	26 (10,548)	0.25% (0.15%-0.34%) ***	26 (8-77) ^{ns}	Metro	1,206 (276,260)	0.44% (0.41%-0.46%) ^{ns}	22 (8-63) ^{ns}
	2nd	96 (22,368)	0.43% (0.34%-0.51%)	21 (5-70)	Urban	124 (29,598)	0.42% (0.35%-0.49%)	19 (6-69)
	3rd	228 (59,305)	0.38% (0.33%-0.43%)	18 (7-69)	Rural	9 (3,998)	0.23% (0.08%-0.37%)	9 (8-15)
	4th	988 (217,524)	0.45% (0.43%-0.48%)	23 (9-61)	Unknown	0 (62)	NA	NA
	Unk	1 (173)	NA	8 (8-8)				
Testis	1st	34 (396)	8.59% (5.83%-11.35%) ***	NA (14-NA) ^{ns}	Metro	1,031 (14,482)	7.12% (6.70%-7.54%) ^{ns}	NA (20-NA) ^{ns}
	2nd	93 (1,146)	8.12% (6.53%-9.70%)	NA (16-NA)	Urban	86 (1,234)	6.97% (5.55%-8.39%)	NA (14-NA)
	3rd	243 (2,715)	8.95% (7.88%-10.02%)	NA (15-NA)	Rural	12 (162)	7.41% (3.37%-11.44%)	NA (13-NA)
	4th	758 (11,592)	6.54% (6.09%-6.99%)	NA (25-NA)	Unknown	0 (3)	NA	NA
	Unk	1 (32)	NA	NA (NA-NA)				
Other GU	1st	15 (346)	4.34% (2.19%-6.48%) ^{ns}	7 (4-16) ^{ns}	Metro	470 (7,647)	6.15% (5.61%-6.68%) ^{ns}	5 (2-12) ^{ns}
	2nd	43 (726)	5.92% (4.21%-7.64%)	6 (2-11)	Urban	49 (933)	5.25% (3.82%-6.68%)	5 (2-11)
	3rd	100 (1,631)	6.13% (4.97%-7.30%)	6 (2-11)	Rural	10 (137)	7.30% (2.94%-11.66%)	4.5 (2-11)
	4th	369 (6,006)	6.14% (5.54%-6.75%)	5 (2-12)	Unknown	0 (1)	NA	NA
	Unk	2 (9)	NA	5.5 (4-7)				
Ovary	1st	42 (790)	5.32% (3.75%-6.88%) *	4 (1-20) ***	Metro	1,532 (26,912)	5.69% (5.42%-5.97%) ^{ns}	16 (2-36) ^{ns}
	2nd	134 (1,941)	6.90% (5.78%-8.03%)	9 (2-27)	Urban	141 (2,542)	5.55% (4.66%-6.44%)	11 (2-25)
	3rd	313 (5,135)	6.10% (5.44%-6.75%)	15 (2-33)	Rural	23 (333)	6.91% (4.18%-9.63%)	11 (3-47)
	4th	1,207 (21,900)	5.51% (5.21%-5.81%)	18 (3-39)	Unknown	0 (2)	NA	NA
	Unk	0 (23)	NA	NA				
Uterus	1st	47 (2,212)	2.12% (1.52%-2.73%) ^{ns}	8 (2-23) ^{ns}	Metro	1,091 (65,544)	1.66% (1.57%-1.76%) ^{ns}	8 (2-21) ^{ns}
	2nd	89 (4,959)	1.79% (1.43%-2.16%)	7 (1-19)	Urban	121 (6,791)	1.78% (1.47%-2.10%)	7 (1-24)
	3rd	237 (12,704)	1.87% (1.63%-2.10%)	9 (2-23)	Rural	20 (1,002)	2.00% (1.13%-2.86%)	16 (6-19)
	4th	858 (53,412)	1.61% (1.50%-1.71%)	8 (2-22)	Unknown	0 (5)	NA	NA
	Unk	1 (55)	NA	NA (NA-NA)				
Cervix	1st	39 (823)	4.74% (3.29%-6.19%) ^{ns}	6 (2-20) ^{ns}	Metro	747 (18,408)	4.06% (3.77%-4.34%) ***	7 (3-16) ^{ns}
	2nd	76 (1,872)	4.06% (3.17%-4.95%)	10 (4-20)	Urban	76 (1,979)	3.84% (2.99%-4.69%)	9 (4-24)
	3rd	174 (3,973)	4.38% (3.74%-5.02%)	6 (3-12)	Rural	21 (271)	7.75% (4.57%-10.93%)	6 (2-12)
	4th	553 (13,961)	3.96% (3.64%-4.28%)	7 (2-18)	Unknown	0 (0)		NA
	Unk	2 (29)	NA	NA (NA-NA)				
Other GYN	1st	15 (460)	3.26% (1.64%-4.88%) ^{ns}	2 (0-5) ^{ns}	Metro	402 (9,123)	4.41% (3.99%-4.83%) ^{ns}	5 (1-23) ^{ns}
	2nd	44 (856)	5.14% (3.66%-6.62%)	7 (1-24)	Urban	52 (1,212)	4.29% (3.15%-5.43%)	5 (1-17)
	3rd	86 (2,008)	4.28% (3.40%-5.17%)	4 (1-32)	Rural	3 (179)	NA	5 (5-NA)
	4th	311 (7,173)	4.34% (3.86%-4.81%)	5 (1-23)	Unknown	0 (0)		NA
	Unk	1 (17)	NA	5 (5-5)				
Bone tumor	1st	20 (146)	13.70% (8.12%-19.28%) ^{ns}	17 (9-50) ^{ns}	Metro	536 (4,582)	11.70% (10.77%-12.63%) ^{ns}	20 (8-NA) ^{ns}
	2nd	44 (344)	12.79% (9.26%-16.32%)	15 (4-32)	Urban	67 (435)	15.40% (12.01%-18.79%)	15 (8-32)
	3rd	123 (933)	13.18% (11.01%-15.35%)	19 (8-NA)	Rural	6 (62)	9.68% (2.32%-17.04%)	NA (14-NA)
	4th	421 (3,646)	11.55% (10.51%-12.58%)	20 (8-NA)	Unknown	0 (0)		NA
	Unk	1 (10)	NA	9 (9-9)				
STS	1st	71 (731)	9.71% (7.57%-11.86%) ^{ns}	7 (2-17) ^{ns}	Metro	1,958 (22,711)	8.62% (8.26%-8.99%) ^{ns}	8 (2-23) ^{ns}
	2nd	161 (1,777)	9.06% (7.73%-10.39%)	10 (4-22)	Urban	195 (2,114)	9.22% (7.99%-10.46%)	9 (3-24)
	3rd	393 (4,342)	9.05% (8.20%-9.90%)	7 (2-21)	Rural	28 (264)	10.61% (6.89%-14.32%)	6.5 (1-14)
	4th	1,556 (18,218)	8.54% (8.14%-8.95%)	8 (2-24)	Unknown	0 (4)	NA	NA
	Unk	0 (25)	NA	NA				
Skin melanoma	1st	32 (2,890)	1.11% (0.73%-1.49%) ^{ns}	11 (1-26) ^{ns}	Metro	948 (95,565)	0.99% (0.93%-1.05%) ^{ns}	6 (2-20) ^{ns}
	2nd	80 (7,005)	1.14% (0.89%-1.39%)	6 (3-14)	Urban	96 (10,243)	0.94% (0.75%-1.12%)	5 (3-25)
	3rd	194 (17,615)	1.10% (0.95%-1.26%)	6 (2-13)	Rural	21 (1,462)	1.44% (0.83%-2.05%)	10 (4-20)
	4th	759 (79,739)	0.95% (0.88%-1.02%)	6 (2-22)	Unknown	0 (17)	NA	NA
	Unk	0 (38)	NA	NA				
Non-skin melanoma	1st	2 (110)	NA ^{ns}	19.5 (1-38) *	Metro	95 (2,707)	3.51% (2.82%-4.20%) ^{ns}	6 (3-13) ^{ns}
	2nd	10 (273)	3.66% (1.43%-5.89%)	10 (4-18)	Urban	11 (334)	3.29% (1.38%-5.21%)	7 (3-18)
	3rd	20 (539)	3.71% (2.11%-5.31%)	4 (2-5)	Rural	0 (42)	NA	NA
	4th	74 (2,158)	3.43% (2.66%-4.20%)	6 (3-15)	Unknown	0 (1)	NA	NA

	Unk	0 (4)	NA	NA				
Skin cancer	1st	0 (180)	NA ^{ns}	NA ^{ns}	Metro	33 (4,775)	0.69% (0.46%-0.93%) ^{ns}	9 (5-17) ^{ns}
	2nd	4 (378)	NA	9 (7-18.5)	Urban	4 (500)	NA	16 (6.5-26)
	3rd	9 (968)	0.93% (0.33%-1.53%)	11 (8-21)	Rural	0 (70)	NA	NA
	4th	24 (3,815)	0.63% (0.38%-0.88%)	8 (5-18)	Unknown	0 (0)		NA
	Unk	0 (4)	NA	NA				
Embryonal tumors	1st	10 (136)	7.35% (2.97%-11.74%) ^{ns}	57 (16-NA) ^{ns}	Metro	305 (3,906)	7.81% (6.97%-8.65%) ^{ns}	NA (26-NA) ^{ns}
	2nd	25 (303)	8.25% (5.15%-11.35%)	NA (23-NA)	Urban	24 (317)	7.57% (4.66%-10.48%)	32 (12-NA)
	3rd	64 (793)	8.07% (6.17%-9.97%)	NA (9-NA)	Rural	5 (57)	8.77% (1.43%-16.12%)	NA (9-NA)
	4th	234 (3,036)	7.71% (6.76%-8.66%)	NA (26-NA)	Unknown	0 (1)	NA	NA
	Unk	1 (13)	NA	NA (NA-NA)				
All other	1st	91 (1,104)	8.24% (6.62%-9.87%) ^{ns}	2 (1-6) ***	Metro	2,600 (29,664)	8.76% (8.44%-9.09%) ^{ns}	5 (1-21) ***
	2nd	217 (2,462)	8.81% (7.69%-9.93%)	4 (1-15)	Urban	254 (2,965)	8.57% (7.56%-9.57%)	4 (1-11)
	3rd	516 (5,787)	8.92% (8.18%-9.65%)	5 (1-16)	Rural	48 (435)	11.03% (8.09%-13.98%)	2 (0-5)
	4th	2,073 (23,679)	8.75% (8.39%-9.11%)	5 (1-21)	Unknown	1 (4)	NA	73 (73-73)
	Unk	6 (36)	16.67% (4.49%-28.84%)	0.5 (0-13)				

[a] The county-level education is presented as quantiles here.

[b] Prevalence was only calculated in category with more than 5 sLM cases.

[c] Survival data (in months) are shown as the median survival and interquartile range in cases with synchronous lung metastasis at diagnosis.

[d] Liver cancer here includes hepatocellular carcinoma and intrahepatic cholangiocarcinoma.

Abbreviations: sLM: synchronous lung metastasis; Unk: unknown; GI: gastrointestinal cancer; GU: genitourinary cancer; GYN: gynecologic cancer; NA: non-applicable; ns: non-significant; *: p<0.05; ***: p<0.001% for intragroup survival comparison by Chi-square tests or log-rank tests.

Supplementary Table S4. Number of cases with synchronous lung metastasis and all cases, prevalence of synchronous lung metastasis and median survival with interquartile range by cancer type and T stage or N stage.

Categories	T stage	No. of sLM (All)	Prevalence [a]	Survival [b]	N stage	No. of sLM (All)	Prevalence [a]	Survival [b]
Head and neck	T1	117 (22,860)	0.51% (0.42%-0.60%) ***	12 (4-29) ***	N0	250 (35,500)	0.70% (0.62%-0.79%) ***	11 (4-30) ***
	T2	272 (18,927)	1.44% (1.27%-1.61%)	10 (4-23)	N1	278 (10,255)	2.71% (2.40%-3.03%)	8 (3-18)
	T3	357 (11,672)	3.06% (2.75%-3.37%)	10 (4-23)	N2	779 (23,828)	3.27% (3.04%-3.50%)	9 (4-20)
	T4	571 (13,187)	4.33% (3.98%-4.68%)	7 (3-16)	N3	165 (2,337)	7.06% (6.02%-8.10%)	8 (3-22)
	Unk	257 (8,251)	3.11% (2.74%-3.49%)	7 (3-20)	Unk	102 (2,977)	3.43% (2.77%-4.08%)	5 (1-15)
Thyroid	T1	46 (44,173)	0.10% (0.07%-0.13%) ***	NA (22-NA) ***	N0	269 (57,001)	0.47% (0.42%-0.53%) ***	23 (3-83) ***
	T2	52 (12,970)	0.40% (0.29%-0.51%)	NA (15-NA)	N1	625 (18,770)	3.33% (3.07%-3.59%)	10 (2-NA)
	T3	211 (15,954)	1.32% (1.15%-1.50%)	83 (43-NA)	N2	0 (0)	NA	NA
	T4	569 (3,012)	18.89% (17.49%-20.29%)	4 (1-22)	N3	0 (0)	NA	NA
	Unk	118 (1,894)	6.23% (5.14%-7.32%)	6 (1-21)	Unk	102 (2,232)	4.57% (3.70%-5.44%)	4 (1-15)
Breast	T1	434 (204,207)	0.21% (0.19%-0.23%) ***	26 (7-65) ***	N0	1,155 (239,387)	0.48% (0.45%-0.51%) ***	18 (3-50) ***
	T2	1,383 (107,635)	1.28% (1.22%-1.35%)	25 (7-52)	N1	2,724 (84,290)	3.23% (3.11%-3.35%)	21 (6-47)
	T3	874 (22,366)	3.91% (3.65%-4.16%)	19 (6-49)	N2	656 (18,768)	3.50% (3.23%-3.76%)	25 (9-55)
	T4	2,446 (15,366)	15.92% (15.34%-16.50%)	18 (5-45)	N3	813 (11,774)	6.91% (6.45%-7.36%)	20 (7-47)
	Unk	835 (9,075)	9.20% (8.61%-9.80%)	14 (1-38)	Unk	624 (4,430)	14.09% (13.06%-15.11%)	10 (1-34)
Main bronchus	T1	18 (413)	4.36% (2.39%-6.33%) ***	10 (3-18) ns	N0	217 (2,190)	9.91% (8.66%-11.16%) ***	4 (1-12) ns
	T2	251 (2,972)	8.45% (7.45%-9.45%)	4 (1-12)	N1	83 (735)	11.29% (9.00%-13.58%)	5 (1-12)
	T3	427 (2,133)	20.02% (18.32%-21.72%)	4 (1-11)	N2	920 (5,334)	17.25% (16.23%-18.26%)	3 (1-9)
	T4	1,074 (4,470)	24.03% (22.77%-25.28%)	3 (1-10)	N3	543 (2,122)	25.59% (23.73%-27.45%)	4 (1-11)
	Unk	83 (890)	9.33% (7.42%-11.24%)	2 (1-9)	Unk	90 (497)	18.11% (14.72%-21.49%)	2 (1-9)
Oesophagus	T1	385 (4,893)	7.87% (7.11%-8.62%) ***	3 (1-9) ***	N0	438 (7,807)	5.61% (5.10%-6.12%) ***	3 (1-9) ns
	T2	54 (1,896)	2.85% (2.10%-3.60%)	8 (3-20)	N1	1,003 (7,966)	12.59% (11.86%-13.32%)	4 (1-10)
	T3	269 (6,564)	4.10% (3.62%-4.58%)	6 (3-12)	N2	136 (2,030)	6.70% (5.61%-7.79%)	5 (2-9)
	T4	405 (2,293)	17.66% (16.10%-19.22%)	4 (1-8)	N3	127 (784)	16.20% (13.62%-18.78%)	3 (1-10)
	Unk	863 (4,422)	19.52% (18.35%-20.68%)	3 (1-9)	Unk	272 (1,481)	18.37% (16.39%-20.34%)	3 (1-8)
Stomach	T1	314 (7,798)	4.03% (3.59%-4.46%) ***	3 (1-9) ***	N0	553 (14,936)	3.70% (3.40%-4.01%) ***	3 (1-10) ***
	T2	64 (2,994)	2.14% (1.62%-2.66%)	4 (1-13)	N1	687 (8,194)	8.38% (7.78%-8.98%)	4 (1-10)
	T3	189 (7,506)	2.52% (2.16%-2.87%)	5 (2-11)	N2	71 (2,834)	2.51% (1.93%-3.08%)	4 (1-12)
	T4	307 (5,568)	5.51% (4.91%-6.11%)	3 (1-7)	N3	90 (2,786)	3.23% (2.57%-3.89%)	3 (1-8)
	Unk	882 (7,959)	11.08% (10.39%-11.77%)	3 (1-9)	Unk	355 (3,075)	11.54% (10.42%-12.67%)	2 (1-7)
Liver [c]	T1	366 (9,114)	4.02% (3.61%-4.42%) ***	2 (1-9) ***	N0	1,023 (18,776)	5.45% (5.12%-5.77%) ***	2 (1-6) ***
	T2	339 (5,456)	6.21% (5.57%-6.85%)	3 (1-8)	N1	503 (2,778)	18.11% (16.67%-19.54%)	3 (1-7)
	T3	540 (5,282)	10.22% (9.41%-11.04%)	2 (1-6)	N2	0 (0)	NA	NA
	T4	187 (1,045)	17.89% (15.57%-20.22%)	2 (0-5)	N3	0 (0)	NA	NA
	Unk	813 (5,370)	15.14% (14.18%-16.10%)	2 (0-5)	Unk	719 (4,713)	15.26% (14.23%-16.28%)	2 (0-5)
Extrahepatic biliary tract	T1	86 (2,257)	3.81% (3.02%-4.60%) ***	4 (2-8) ***	N0	250 (7,479)	3.34% (2.94%-3.75%) ***	3 (1-8) ***
	T2	52 (3,278)	1.59% (1.16%-2.01%)	4 (1-9)	N1	209 (3,948)	5.29% (4.60%-5.99%)	4 (1-11)
	T3	176 (3,891)	4.52% (3.87%-5.18%)	4 (1-11)	N2	62 (522)	11.88% (9.10%-14.65%)	3 (1-8)
	T4	66 (1,401)	4.71% (3.60%-5.82%)	4 (1-11)	N3	0 (0)	NA	NA
	Unk	496 (3,411)	14.54% (13.36%-15.72%)	2 (1-7)	Unk	355 (2,289)	15.51% (14.03%-16.99%)	2 (1-6)
Pancreas	T1	138 (2,811)	4.91% (4.11%-5.71%) ***	3 (1-10) ***	N0	2,343 (27,839)	8.42% (8.09%-8.74%) ***	3 (1-8) ***
	T2	1,394 (11,490)	12.13% (11.54%-12.73%)	3 (1-7)	N1	2,297 (19,150)	11.99% (11.53%-12.45%)	3 (1-8)
	T3	1,425 (20,240)	7.04% (6.69%-7.39%)	3 (1-9)	N2	0 (0)	NA	NA
	T4	1,219 (9,886)	12.33% (11.68%-12.98%)	4 (1-10)	N3	0 (0)	NA	NA
	Unk	1,506 (8,015)	18.79% (17.93%-19.64%)	2 (1-5)	Unk	1,042 (5,453)	19.11% (18.07%-20.15%)	2 (1-5)
Small intestine	T1	28 (1,595)	1.76% (1.11%-2.40%) ***	6 (2-11) ***	N0	93 (4,591)	2.03% (1.62%-2.43%) ***	7 (2-17) *
	T2	3 (1,277)	NA	12.5 (11-NA)	N1	87 (4,064)	2.14% (1.70%-2.59%)	10 (2-43)
	T3	39 (2,870)	1.36% (0.94%-1.78%)	19 (8-NA)	N2	21 (429)	4.90% (2.85%-6.94%)	6 (1-9)
	T4	63 (2,363)	2.67% (2.02%-3.32%)	6 (1-17)	N3	0 (0)	NA	NA
	Unk	106 (1,523)	6.96% (5.68%-8.24%)	7 (1-14)	Unk	38 (544)	6.99% (4.84%-9.13%)	10 (1-35)

Colon & rectum	T1	1,047 (34,737)	3.01% (2.83%-3.19%) ***	9 (2-23) ***	N0	3,235 (106,169)	3.05% (2.94%-3.15%) ***	10 (2-24) ***
	T2	175 (22,176)	0.79% (0.67%-0.91%)	19 (5-36)	N1	3,236 (48,113)	6.73% (6.50%-6.95%) ***	14 (4-28) ***
	T3	2,563 (83,101)	3.08% (2.97%-3.20%)	19 (7-37)	N2	1,586 (24,699)	6.42% (6.12%-6.73%)	14 (5-29)
	T4	2,122 (28,487)	7.45% (7.14%-7.75%)	11 (4-25)	N3	0 (0)	NA	NA
	Unk	3,738 (18,038)	20.72% (20.13%-21.31%)	7 (2-18)	Unk	1,588 (7,558)	21.01% (20.09%-21.93%)	7 (2-18)
Right colon	T1	314 (9,673)	3.25% (2.89%-3.60%) ***	6 (1-16) ***	N0	789 (41,238)	1.91% (1.78%-2.05%) ***	7 (2-19) ***
	T2	31 (9,563)	0.32% (0.21%-0.44%)	6 (3-28)	N1	955 (17,935)	5.32% (5.00%-5.65%)	9 (2-22)
	T3	742 (35,435)	2.09% (1.94%-2.24%)	15 (5-30)	N2	672 (11,433)	5.88% (5.45%-6.31%)	11 (3-24)
	T4	730 (13,174)	5.54% (5.15%-5.93%)	9 (3-20)	N3	0 (0)	NA	NA
	Unk	960 (4,538)	21.15% (19.97%-22.34%)	5 (1-14)	Unk	361 (1,777)	20.32% (18.44%-22.19%)	4 (1-12)
Left colon	T1	351 (13,064)	2.69% (2.41%-2.96%) ***	10 (2-23) ***	N0	1,224 (36,095)	3.39% (3.20%-3.58%) ***	12 (3-26) ***
	T2	49 (7,142)	0.69% (0.49%-0.88%)	23 (10-56)	N1	1,048 (16,814)	6.23% (5.87%-6.60%)	16 (5-31)
	T3	870 (27,881)	3.12% (2.92%-3.32%)	21 (8-40)	N2	585 (8,820)	6.63% (6.11%-7.15%)	17 (7-33)
	T4	861 (10,637)	8.09% (7.58%-8.61%)	14 (4-28)	N3	0 (0)	NA	NA
	Unk	1,239 (5,290)	23.42% (22.28%-24.56%)	9 (2-21)	Unk	513 (2,285)	22.45% (20.74%-24.16%)	7 (2-18)
Unspecified colon	T1	33 (368)	8.97% (6.05%-11.89%) ***	2 (1-10) ***	N0	274 (2,257)	12.14% (10.79%-13.49%) ***	3 (1-10) ns
	T2	2 (196)	NA	2.5 (1-4)	N1	137 (866)	15.82% (13.39%-18.25%)	4 (1-15)
	T3	50 (1,208)	4.14% (3.02%-5.26%)	12 (5-28)	N2	40 (550)	7.27% (5.10%-9.44%)	10 (3-20)
	T4	85 (887)	9.58% (7.65%-11.52%)	7 (1-18)	N3	0 (0)	NA	NA
	Unk	576 (2,068)	27.85% (25.92%-29.79%)	3 (1-10)	Unk	295 (1,054)	27.99% (25.28%-30.70%)	3 (1-12)
Rectum	T1	349 (11,632)	3.00% (2.69%-3.31%) ***	14 (4-26) ***	N0	948 (26,579)	3.57% (3.34%-3.79%) ***	14 (4-29) ***
	T2	93 (5,275)	1.76% (1.41%-2.12%)	21 (7-36)	N1	1,096 (12,498)	8.77% (8.27%-9.27%)	17 (7-31)
	T3	901 (18,577)	4.85% (4.54%-5.16%)	21 (9-40)	N2	289 (3,896)	7.42% (6.59%-8.24%)	19 (9-40)
	T4	446 (3,789)	11.77% (10.74%-12.80%)	13 (5-28)	N3	0 (0)	NA	NA
	Unk	963 (6,142)	15.68% (14.77%-16.59%)	12 (3-24)	Unk	419 (2,442)	17.16% (15.66%-18.65%)	12 (3-22)
Anus	T1	6 (1,940)	0.31% (0.06%-0.56%) ***	23 (13-35) ns	N0	59 (6,054)	0.97% (0.73%-1.22%) ***	14 (7-29) ns
	T2	39 (3,380)	1.15% (0.79%-1.51%)	13 (6-36)	N1	40 (933)	4.29% (2.99%-5.59%)	9 (3-21)
	T3	45 (1,471)	3.06% (2.18%-3.94%)	10 (6-16)	N2	33 (1,124)	2.94% (1.95%-3.92%)	11 (5-20)
	T4	31 (789)	3.93% (2.57%-5.28%)	12 (5-21)	N3	33 (843)	3.91% (2.61%-5.22%)	13 (6-NA)
	Unk	62 (1,825)	3.40% (2.57%-4.23%)	10 (4-20)	Unk	18 (451)	3.99% (2.18%-5.80%)	6 (4-14)
Other GI	T1	0 (2,114)	NA***	NA ***	N0	24 (4,690)	0.51% (0.31%-0.72%) ***	36 (8-NA) ***
	T2	0 (459)	NA	NA	N1	14 (694)	2.02% (0.97%-3.06%)	5 (3-18)
	T3	8 (1,347)	0.59% (0.18%-1.00%)	22 (7-36)	N2	5 (330)	1.52% (0.20%-2.83%)	13 (12-22)
	T4	29 (1,900)	1.53% (0.98%-2.08%)	14 (4-NA)	N3	0 (0)	NA	NA
	Unk	643 (2,791)	23.04% (21.48%-24.60%)	2 (0-6)	Unk	637 (2,897)	21.99% (20.48%-23.50%)	2 (0-5)
Kidney	T1	753 (44,005)	1.71% (1.59%-1.83%) ***	7 (2-22) ***	N0	3,164 (62,789)	5.04% (4.87%-5.21%) ***	11 (3-30) ***
	T2	1,064 (7,771)	13.69% (12.93%-14.46%)	8 (3-21)	N1	1,938 (4,290)	45.17% (43.69%-46.66%)	5 (2-13)
	T3	2,267 (13,592)	16.68% (16.05%-17.31%)	12 (4-30)	N2	0 (0)	NA	NA
	T4	810 (1,747)	46.37% (44.03%-48.70%)	5 (2-12)	N3	0 (0)	NA	NA
	Unk	929 (2,490)	37.31% (35.41%-39.21%)	4 (1-11)	Unk	721 (2,526)	28.54% (26.78%-30.30%)	4 (2-13)
Bladder	T1	192 (20,605)	0.93% (0.80%-1.06%) ***	5 (1-13) ***	N0	641 (36,071)	1.78% (1.64%-1.91%) ***	4 (1-11) ***
	T2	465 (12,643)	3.68% (3.35%-4.01%)	5 (2-11)	N1	132 (1,568)	8.42% (7.04%-9.79%)	4 (1-10)
	T3	110 (3,824)	2.88% (2.35%-3.41%)	5 (3-13)	N2	199 (2,033)	9.79% (8.50%-11.08%)	5 (2-11)
	T4	214 (2,874)	7.45% (6.49%-8.41%)	4 (2-11)	N3	57 (538)	10.59% (7.99%-13.20%)	5 (2-12)
	Unk	234 (1,722)	13.59% (11.97%-15.21%)	2 (1-6)	Unk	186 (1,458)	12.76% (11.04%-14.47%)	3 (1-6)
Prostate	T1	259 (127,901)	0.20% (0.18%-0.23%) ***	30 (11-72) ***	N0	567 (287,587)	0.20% (0.18%-0.21%) ***	25 (9-63) ns
	T2	316 (133,949)	0.24% (0.21%-0.26%)	24 (10-65)	N1	489 (11,121)	4.40% (4.02%-4.78%)	19 (8-53)
	T3	136 (35,776)	0.38% (0.32%-0.44%)	26 (9-NA)	N2	0 (0)	NA	NA
	T4	218 (3,431)	6.35% (5.54%-7.17%)	15 (5-37)	N3	0 (0)	NA	NA
	Unk	410 (8,861)	4.63% (4.19%-5.06%)	18 (7-47)	Unk	283 (11,210)	2.52% (2.23%-2.81%)	22 (7-NA)
Testis	T1	347 (9,864)	3.52% (3.15%-3.88%) ***	NA (28-NA) ***	N0	359 (11,797)	3.04% (2.73%-3.35%) ***	NA (23-NA) ***
	T2	343 (4,265)	8.04% (7.23%-8.86%)	NA (62-NA)	N1	315 (1,469)	21.44% (19.34%-23.54%)	NA (16-NA)
	T3	231 (739)	31.26% (27.92%-34.60%)	NA (52-NA)	N2	164 (1,003)	16.35% (14.06%-18.64%)	NA (NA-NA)
	T4	19 (90)	21.11% (12.68%-29.54%)	NA (7-NA)	N3	198 (851)	23.27% (20.43%-26.11%)	NA (23-NA)
	Unk	189 (923)	20.48% (17.87%-23.08%)	23 (6-NA)	Unk	93 (761)	12.22% (9.89%-14.55%)	47 (8-NA)
Other GU	T1	66 (2,910)	2.27% (1.73%-2.81%) ***	5 (2-14) ns	N0	148 (6,161)	2.40% (2.02%-2.78%) ***	7 (3-14) ns

	T2	24 (1,391)	1.73% (1.04%-2.41%)	6 (2-12)	N1	108 (658)	16.41% (13.58%-19.24%)	5 (1-12)
	T3	114 (2,402)	4.75% (3.90%-5.60%)	8 (2-14)	N2	121 (762)	15.88% (13.28%-18.47%)	6 (2-11)
	T4	121 (745)	16.24% (13.59%-18.89%)	5 (2-10)	N3	14 (170)	8.24% (4.10%-12.37%)	7 (2-12)
	Unk	204 (1,270)	16.06% (14.04%-18.08%)	4 (1-11)	Unk	138 (967)	14.27% (12.07%-16.48%)	3 (1-10)
Ovary	T1	80 (8,707)	0.92% (0.72%-1.12%) ***	12 (1-43) ***	N0	787 (20,924)	3.76% (3.50%-4.02%) ***	20 (4-42) ***
	T2	158 (3,847)	4.11% (3.48%-4.73%)	13 (4-38)	N1	522 (6,245)	8.36% (7.67%-9.05%)	18 (4-37)
	T3	1,019 (15,205)	6.70% (6.30%-7.10%)	21 (5-41)	N2	0 (0)	NA	NA
	T4	NA ()	#VALUE!	NA	N3	0 (0)	NA	NA
	Unk	439 (2,030)	21.63% (19.83%-23.42%)	4 (1-21)	Unk	387 (2,620)	14.77% (13.41%-16.13%)	5 (1-21)
Uterus	T1	185 (58,868)	0.31% (0.27%-0.36%) ***	10 (3-27) ***	N0	479 (64,137)	0.75% (0.68%-0.81%) ***	11 (3-30) ***
	T2	90 (4,585)	1.96% (1.56%-2.36%)	13 (5-32)	N1	305 (4,309)	7.08% (6.31%-7.84%)	8 (2-18)
	T3	527 (6,832)	7.71% (7.08%-8.35%)	9 (3-23)	N2	270 (2,784)	9.70% (8.60%-10.80%)	7 (1-15)
	T4	125 (949)	13.17% (11.02%-15.32%)	9 (2-23)	N3	0 (0)	NA	NA
	Unk	305 (2,108)	14.47% (12.97%-15.97%)	4 (1-12)	Unk	178 (2,112)	8.43% (7.24%-9.61%)	5 (1-17)
Cervix	T1	93 (11,413)	0.81% (0.65%-0.98%) ***	10 (3-20) ***	N0	227 (14,600)	1.55% (1.35%-1.76%) ***	9 (3-20) *
	T2	142 (4,530)	3.13% (2.63%-3.64%)	9 (3-25)	N1	487 (5,254)	9.27% (8.48%-10.05%)	7 (3-16)
	T3	333 (3,173)	10.49% (9.43%-11.56%)	7 (3-18)	N2	0 (0)	NA	NA
	T4	110 (778)	14.14% (11.69%-16.59%)	7 (3-14)	N3	0 (0)	NA	NA
	Unk	166 (764)	21.73% (18.80%-24.65%)	4 (1-12)	Unk	130 (804)	16.17% (13.62%-18.71%)	5 (1-17)
Other GYN	T1	49 (5,596)	0.88% (0.63%-1.12%) ***	6 (2-42) ***	N0	114 (7,185)	1.59% (1.30%-1.88%) ***	14 (2-54) ***
	T2	35 (1,455)	2.41% (1.62%-3.19%)	8 (2-32)	N1	110 (1,557)	7.06% (5.79%-8.34%)	6 (1-18)
	T3	138 (2,073)	6.66% (5.58%-7.73%)	11 (2-47)	N2	58 (614)	9.45% (7.13%-11.76%)	2 (1-8)
	T4	36 (221)	16.29% (11.42%-21.16%)	3 (1-9)	N3	10 (93)	10.75% (4.46%-17.05%)	3 (2-11)
	Unk	199 (1,169)	17.02% (14.87%-19.18%)	3 (0-13)	Unk	165 (1,065)	15.49% (13.32%-17.67%)	3 (1-15)
Bone	T1	94 (2,058)	4.57% (3.67%-5.47%) ***	30 (9-NA) ***	N0	459 (4,472)	10.26% (9.37%-11.15%) ***	20 (9-NA) ns
	T2	337 (1,983)	16.99% (15.34%-18.65%)	21 (9-NA)	N1	84 (167)	50.30% (42.72%-57.88%)	15 (7-42)
	T3	44 (126)	34.92% (26.60%-43.24%)	13 (7-35)	N2	0 (0)	NA	NA
	T4	NA ()	#VALUE!	NA	N3	0 (0)	NA	NA
	Unk	134 (912)	14.69% (12.40%-16.99%)	12 (4-36)	Unk	66 (440)	15.00% (11.66%-18.34%)	18 (4-48)
STS	T1	265 (5,651)	4.69% (4.14%-5.24%) ***	14 (5-32) ***	N0	1,280 (16,937)	7.56% (7.16%-7.96%) ***	11 (3-25) ***
	T2	1,013 (10,654)	9.51% (8.95%-10.07%)	11 (3-27)	N1	313 (1,080)	28.98% (26.28%-31.69%)	6 (2-20)
	T3	64 (300)	21.33% (16.70%-25.97%)	7 (2-14)	N2	0 (5)	NA	NA
	T4	46 (156)	29.49% (22.33%-36.64%)	4 (1-14)	N3	0 (0)	NA	NA
	Unk	793 (8,332)	9.52% (8.89%-10.15%)	5 (1-17)	Unk	588 (7,071)	8.32% (7.67%-8.96%)	5 (1-19)
Skin melanoma	T1	63 (70,613)	0.09% (0.07%-0.11%) ***	11 (4-26) ***	N0	362 (95,447)	0.38% (0.34%-0.42%) ***	8 (3-29) ***
	T2	57 (15,179)	0.38% (0.28%-0.47%)	11 (5-21)	N1	195 (4,665)	4.18% (3.61%-4.75%)	6 (2-18)
	T3	88 (8,862)	0.99% (0.79%-1.20%)	13 (6-48)	N2	61 (2,219)	2.75% (2.07%-3.43%)	11 (4-33)
	T4	211 (6,773)	3.12% (2.70%-3.53%)	7 (3-24)	N3	95 (1,266)	7.50% (6.05%-8.96%)	7 (2-19)
	Unk	646 (5,860)	11.02% (10.22%-11.83%)	4 (2-14)	Unk	352 (3,690)	9.54% (8.59%-10.49%)	4 (2-12)
Non-skin melanoma	T1	5 (601)	0.83% (0.11%-1.56%) ***	27 (10-27) ns	N0	18 (2,187)	0.82% (0.44%-1.20%) ***	10 (5-NA) ***
	T2	1 (467)	NA	NA (NA-NA)	N1	11 (124)	8.87% (3.87%-13.88%)	10 (4-27)
	T3	4 (629)	NA	6 (4-10.5)	N2	0 (31)	NA	NA
	T4	11 (473)	2.33% (0.97%-3.68%)	7 (1-NA)	N3	1 (21)	NA	1 (1-1)
	Unk	85 (914)	9.30% (7.42%-11.18%)	5 (3-12)	Unk	76 (721)	10.54% (8.30%-12.78%)	5 (3-9)
Skin cancer	T1	5 (2,125)	0.24% (0.03%-0.44%) ***	16.5 (6-26) ns	N0	15 (4,118)	0.36% (0.18%-0.55%) ***	11 (6-25) ns
	T2	11 (1,080)	1.02% (0.42%-1.62%)	10 (7-12)	N1	8 (729)	1.10% (0.34%-1.85%)	8 (7-18)
	T3	2 (200)	NA	5 (3-7)	N2	6 (111)	5.41% (1.20%-9.61%)	10 (8-17)
	T4	7 (119)	5.88% (1.65%-10.11%)	17 (8-18)	N3	0 (3)	NA	NA
	Unk	12 (1,821)	0.66% (0.29%-1.03%)	9 (5-20)	Unk	8 (384)	2.08% (0.65%-3.51%)	5 (4-34)
Embryonal tumors	T1	2 (287)	NA***	9.5 (5-NA) *	N0	12 (677)	1.77% (0.78%-2.77%) ***	11.5 (6-NA) ***
	T2	13 (413)	3.15% (1.46%-4.83%)	13 (9-57)	N1	12 (195)	6.15% (2.78%-9.53%)	14 (7-20)
	T3	0 (68)	NA	NA	N2	0 (1)	NA	NA
	T4	0 (28)	NA	NA	N3	0 (0)	NA	NA

	Unk	319 (3,485)	9.15% (8.20%-10.11%)	NA (23-NA)	Unk	310 (3,408)	9.10% (8.13%-10.06%)	NA (33-NA)
All other	T1	175 (5,715)	3.06% (2.62%-3.51%) ***	15 (4-NA) ***	N0	655 (16,693)	3.92% (3.63%-4.22%) ***	9 (2-23) ***
	T2	245 (4,476)	5.47% (4.81%-6.14%)	9 (2-23)	N1	378 (2,579)	14.66% (13.29%-16.02%)	6 (1-20)
	T3	431 (6,823)	6.32% (5.74%-6.89%)	10 (2-29)	N2	165 (1,404)	11.75% (10.07%-13.44%)	4 (1-13)
	T4	308 (2,616)	11.77% (10.54%-13.01%)	4 (1-12)	N3	27 (141)	19.15% (12.65%-25.64%)	3 (1-8)
	Unk	1,744 (13,438)	12.98% (12.41%-13.55%)	3 (0-16)	Unk	1,678 (12,251)	13.70% (13.09%-14.31%)	3 (0-20)

[a] Prevalence was only calculated in category with more than 5 sLM cases.

[b] Survival data (in months) are shown as the median survival and interquartile range in cases with synchronous lung metastasis at diagnosis.

[c] Liver cancer here includes hepatocellular carcinoma and intrahepatic cholangiocarcinoma.

Abbreviations: sLM: synchronous lung metastasis; Unk: unknown; GI: gastrointestinal cancer; GU: genitourinary cancer; GYN: gynecologic cancer; STS: soft-tissue sarcoma; NA: non-applicable; ns: non-significant; *: p<0.05; ***: p<0.001% for intragroup survival comparison by Chi-square tests or log-rank tests.

Supplementary Table S5. Number of cases with synchronous lung metastasis and all cases, prevalence of synchronous lung metastasis and median survival with interquartile range by cancer type and insurance status or marital status.

Categories	Insurance	No. of sLM (All)	Prevalence [a]	Survival [b]	Marriage	No. of sLM (All)	Prevalence [a]	Survival [b]
Brain	Insured	16 (26,006)	0.06% (0.03%-0.09%) [ns]	8 (2.5-23) [ns]	Married	6 (14,987)	0.04% (0.01%-0.07%) [ns]	8 (4-11) [ns]
	Uninsured	1 (1,028)	NA	16 (16-16)	Unmarried	10 (11,351)	0.09% (0.03%-0.14%)	9.5 (2-32)
	Unk	0 (451)	NA	NA	Unk	1 (1,147)	NA	NA (NA-NA)
Head and neck	Insured	1,436 (69,299)	2.07% (1.97%-2.18%) [***]	9 (3-22) [ns]	Married	628 (38,285)	1.64% (1.51%-1.77%) [***]	10 (4-24) [***]
	Uninsured	107 (3,087)	3.47% (2.82%-4.11%)	7 (2-15)	Unmarried	864 (30,943)	2.79% (2.61%-2.98%)	8 (3-18)
	Unk	31 (2,511)	1.23% (0.80%-1.67%)	7 (4-17)	Unk	82 (5,669)	1.45% (1.14%-1.76%)	8 (3-21)
Thyroid	Insured	947 (74,183)	1.28% (1.20%-1.36%) [ns]	11 (2-79) [ns]	Married	509 (46,261)	1.10% (1.01%-1.20%) [***]	10 (2-70) [ns]
	Uninsured	34 (2,016)	1.69% (1.12%-2.25%)	25 (3-NA)	Unmarried	450 (26,865)	1.68% (1.52%-1.83%)	11 (2-NA)
	Unk	15 (1,804)	0.83% (0.41%-1.25%)	2 (0.5-NA)	Unk	37 (4,877)	0.76% (0.52%-1.00%)	20 (5-NA)
Breast	Insured	5,515 (345,324)	1.60% (1.56%-1.64%) [***]	20 (5-48) [***]	Married	2,329 (196,295)	1.19% (1.14%-1.23%) [***]	26 (8-58) [***]
	Uninsured	321 (6,149)	5.22% (4.66%-5.78%)	14 (2-40)	Unmarried	3,310 (142,801)	2.32% (2.24%-2.40%)	16 (3-41)
	Unk	136 (7,176)	1.90% (1.58%-2.21%)	22 (4-50)	Unk	333 (19,553)	1.70% (1.52%-1.88%)	24 (5-52)
Main bronchus	Insured	1,744 (10,230)	17.05% (16.32%-17.78%) [ns]	4 (1-10) [ns]	Married	876 (5,128)	17.08% (16.05%-18.11%) [ns]	4 (1-12) [***]
	Uninsured	85 (491)	17.31% (13.96%-20.66%)	2 (1-8)	Unmarried	901 (5,337)	16.88% (15.88%-17.89%)	3 (1-9)
	Unk	24 (157)	15.29% (9.66%-20.92%)	2 (0-8)	Unk	76 (413)	18.40% (14.66%-22.14%)	3 (1-9)
Oesophagus	Insured	1,838 (18,911)	9.72% (9.30%-10.14%) [***]	4 (1-10) [ns]	Married	986 (10,720)	9.20% (8.65%-9.74%) [***]	5 (1-11) [***]
	Uninsured	93 (687)	13.54% (10.98%-16.10%)	3 (1-6)	Unmarried	898 (8,235)	10.90% (10.23%-11.58%)	3 (1-8)
	Unk	45 (470)	9.57% (6.91%-12.23%)	3 (0.5-8)	Unk	92 (1,113)	8.27% (6.65%-9.88%)	5 (1-12)
Stomach	Insured	1,619 (29,790)	5.43% (5.18%-5.69%) [***]	3 (1-9) [ns]	Married	978 (17,582)	5.56% (5.22%-5.90%) [***]	4 (1-10) [***]
	Uninsured	101 (1,248)	8.09% (6.58%-9.61%)	3 (1-8)	Unmarried	715 (12,469)	5.73% (5.33%-6.14%)	3 (1-8)
	Unk	36 (787)	4.57% (3.11%-6.03%)	2 (1-14)	Unk	63 (1,774)	3.55% (2.69%-4.41%)	3 (1-11)
Liver [c]	Insured	2,083 (24,796)	8.40% (8.06%-8.75%) [***]	2 (1-6) [*]	Married	1,160 (13,739)	8.44% (7.98%-8.91%) [*]	3 (1-7) [***]
	Uninsured	119 (948)	12.55% (10.44%-14.66%)	1 (0-4)	Unmarried	997 (11,212)	8.89% (8.37%-9.42%)	2 (0-5)
	Unk	43 (523)	8.22% (5.87%-10.58%)	3 (1-7)	Unk	88 (1,316)	6.69% (5.34%-8.04%)	3 (1-10)
Extrahepatic biliary tract	Insured	817 (13,568)	6.02% (5.62%-6.42%) [***]	3 (1-8) [ns]	Married	435 (7,541)	5.77% (5.24%-6.29%) [ns]	4 (1-9) [*]
	Uninsured	36 (458)	7.86% (5.40%-10.32%)	2 (0.5-7)	Unmarried	405 (6,068)	6.67% (6.05%-7.30%)	2 (1-7)
	Unk	23 (212)	10.85% (6.66%-15.04%)	2 (1-6)	Unk	36 (629)	5.72% (3.91%-7.54%)	3 (1-9)
Pancreas	Insured	5,398 (50,109)	10.77% (10.50%-11.04%) [ns]	3 (1-8) [ns]	Married	3,043 (29,231)	10.41% (10.06%-10.76%) [***]	3 (1-9) [***]
	Uninsured	183 (1,523)	12.02% (10.38%-13.65%)	2 (1-5)	Unmarried	2,385 (20,874)	11.43% (10.99%-11.86%)	2 (1-6)
	Unk	101 (810)	12.47% (10.19%-14.74%)	2 (0-6)	Unk	254 (2,337)	10.87% (9.61%-12.13%)	2 (1-6)
Small intestine	Insured	225 (9,141)	2.46% (2.14%-2.78%) [ns]	8 (2-22) [*]	Married	118 (5,507)	2.14% (1.76%-2.53%) [*]	11 (4-35) [***]
	Uninsured	9 (247)	3.64% (1.31%-5.98%)	4 (2-8)	Unmarried	104 (3,530)	2.95% (2.39%-3.50%)	6 (1-14)
	Unk	5 (240)	2.08% (0.28%-3.89%)	2 (1-2)	Unk	17 (591)	2.88% (1.53%-4.22%)	4 (1-8)
Colon & rectum	Insured	8,918 (175,294)	5.09% (4.98%-5.19%) [***]	11 (3-25) [ns]	Married	4,546 (96,321)	4.72% (4.59%-4.85%) [***]	14 (4-28) [***]
	Uninsured	547 (6,268)	8.73% (8.03%-9.43%)	11 (3-25)	Unmarried	4,564 (78,491)	5.81% (5.65%-5.98%)	9 (2-23)
	Unk	180 (4,977)	3.62% (3.10%-4.14%)	10 (1-28)	Unk	535 (11,727)	4.56% (4.18%-4.94%)	9 (2-26)
Right colon	Insured	2,600 (69,097)	3.76% (3.62%-3.90%) [***]	8 (2-21) [*]	Married	1,331 (36,427)	3.65% (3.46%-3.85%) [*]	10 (3-22) [***]
	Uninsured	127 (1,927)	6.59% (5.48%-7.70%)	9 (2-20)	Unmarried	1,285 (32,074)	4.01% (3.79%-4.22%)	6 (2-19)
	Unk	50 (1,359)	3.68% (2.68%-4.68%)	2 (0.5-11)	Unk	161 (3,882)	4.15% (3.52%-4.77%)	9 (2-21)

Left colon	Insured	3,112 (59,815)	5.20% (5.02%-5.38%) ***	14 (4-28) ns	Married	1,663 (33,713)	4.93% (4.70%-5.16%) ***	15 (5-31) ***
	Uninsured	208 (2,470)	8.42% (7.33%-9.52%)	11 (3-26)	Unmarried	1,521 (26,194)	5.81% (5.52%-6.09%)	12 (3-24)
	Unk	50 (1,729)	2.89% (2.10%-3.68%)	16 (3-32)	Unk	186 (4,107)	4.53% (3.89%-5.16%)	8 (2-24)
Unspecified colon	Insured	676 (4,294)	15.74% (14.65%-16.83%) ***	3 (1-12) ns	Married	306 (2,108)	14.52% (13.01%-16.02%) *	4 (1-15) ***
	Uninsured	46 (212)	21.70% (16.15%-27.25%)	4 (1-10)	Unmarried	395 (2,279)	17.33% (15.78%-18.89%)	3 (1-10)
	Unk	24 (221)	10.86% (6.76%-14.96%)	3 (1-14)	Unk	45 (340)	13.24% (9.63%-16.84%)	4 (0.5-15)
Rectum	Insured	2,530 (42,088)	6.01% (5.78%-6.24%) ***	15 (5-30) ns	Married	1,246 (24,073)	5.18% (4.90%-5.46%) ***	18 (7-33) ***
	Uninsured	166 (1,659)	10.01% (8.56%-11.45%)	14 (5-27)	Unmarried	1,363 (17,944)	7.60% (7.21%-7.98%)	13 (4-26)
	Unk	56 (1,668)	3.36% (2.49%-4.22%)	20 (4-32)	Unk	143 (3,398)	4.21% (3.53%-4.88%)	16 (4-37)
Anus	Insured	170 (8,789)	1.93% (1.65%-2.22%) ns	11 (5-22) ns	Married	62 (3,693)	1.68% (1.26%-2.09%) ns	14 (7-24) ns
	Uninsured	9 (370)	2.43% (0.86%-4.00%)	46 (5-46)	Unmarried	107 (5,118)	2.09% (1.70%-2.48%)	10 (5-17)
	Unk	4 (246)	NA	12 (1-12)	Unk	14 (594)	2.36% (1.14%-3.58%)	6 (5-17)
Other GI	Insured	648 (8,045)	8.05% (7.46%-8.65%) ns	2 (0-7) ns	Married	328 (4,440)	7.39% (6.62%-8.16%) ns	2 (0-9) ***
	Uninsured	21 (336)	6.25% (3.66%-8.84%)	2 (1-5)	Unmarried	319 (3,737)	8.54% (7.64%-9.43%)	1 (0-4)
	Unk	11 (230)	4.78% (2.02%-7.54%)	2 (1-5)	Unk	33 (434)	7.60% (5.11%-10.10%)	2 (1-5)
Kidney	Insured	5,506 (66,325)	8.30% (8.09%-8.51%) ***	8 (2-22) ns	Married	3,409 (41,424)	8.23% (7.96%-8.49%) ***	8 (3-25) ***
	Uninsured	252 (2,039)	12.36% (10.93%-13.79%)	7 (2-19)	Unmarried	2,196 (24,399)	9.00% (8.64%-9.36%)	6 (2-18)
	Unk	65 (1,241)	5.24% (4.00%-6.48%)	6 (2-23)	Unk	218 (3,782)	5.76% (5.02%-6.51%)	11 (2-26)
Bladder	Insured	1,167 (39,448)	2.96% (2.79%-3.13%) ***	4 (1-11) ns	Married	575 (23,066)	2.49% (2.29%-2.69%) ***	5 (2-12) ***
	Uninsured	31 (860)	3.60% (2.36%-4.85%)	4 (1-10)	Unmarried	593 (15,914)	3.73% (3.43%-4.02%)	3 (1-9)
	Unk	17 (1,360)	1.25% (0.66%-1.84%)	2 (1-4)	Unk	47 (2,688)	1.75% (1.25%-2.24%)	2 (1-9)
Prostate	Insured	1,244 (271,303)	0.46% (0.43%-0.48%) ***	22 (8-63) ns	Married	736 (194,927)	0.38% (0.35%-0.40%) ***	22 (8-63) ns
	Uninsured	62 (4,571)	1.36% (1.02%-1.69%)	26 (9-NA)	Unmarried	525 (68,084)	0.77% (0.71%-0.84%)	23 (9-67)
	Unk	33 (34,044)	0.10% (0.06%-0.13%)	21 (4-NA)	Unk	78 (46,907)	0.17% (0.13%-0.20%)	18 (10-48)
Testis	Insured	1,002 (13,977)	7.17% (6.74%-7.60%) ***	NA (24-NA) ***	Married	267 (6,217)	4.29% (3.79%-4.80%) ***	NA (19-NA) ns
	Uninsured	117 (1,245)	9.40% (7.78%-11.02%)	NA (10-NA)	Unmarried	829 (8,589)	9.65% (9.03%-10.28%)	NA (21-NA)
	Unk	10 (659)	1.52% (0.58%-2.45%)	NA (NA-NA)	Unk	33 (1,075)	3.07% (2.04%-4.10%)	NA (NA-NA)
Other GU	Insured	508 (8,194)	6.20% (5.68%-6.72%) ns	5 (2-12) ns	Married	286 (4,796)	5.96% (5.29%-6.63%) *	6 (2-13) ***
	Uninsured	6 (232)	2.59% (0.54%-4.63%)	3 (1-6)	Unmarried	220 (3,321)	6.62% (5.78%-7.47%)	4 (2-10)
	Unk	15 (292)	5.14% (2.60%-7.67%)	7 (2-13)	Unk	23 (601)	3.83% (2.29%-5.36%)	10 (2-15)
Ovary	Insured	1,604 (28,263)	5.68% (5.41%-5.95%) ns	16 (3-36) ns	Married	768 (14,485)	5.30% (4.94%-5.67%) ***	21 (4-45) ***
	Uninsured	67 (1,067)	6.28% (4.82%-7.73%)	13 (1-27)	Unmarried	863 (13,959)	6.18% (5.78%-6.58%)	10 (2-32)
	Unk	25 (459)	5.45% (3.37%-7.52%)	8 (2-23)	Unk	65 (1,345)	4.83% (3.69%-5.98%)	7 (2-23)
Cervix	Insured	1,141 (69,824)	1.63% (1.54%-1.73%) ***	8 (2-22) ns	Married	512 (37,005)	1.38% (1.26%-1.50%) ***	9 (2-25) ns
	Uninsured	67 (2,167)	3.09% (2.36%-3.82%)	7 (1-26)	Unmarried	663 (32,111)	2.06% (1.91%-2.22%)	7 (2-20)
	Unk	24 (1,351)	1.78% (1.07%-2.48%)	3 (1-7)	Unk	57 (4,226)	1.35% (1.00%-1.70%)	6 (1-20)
Uterus	Insured	765 (18,755)	4.08% (3.80%-4.36%) ns	7 (3-17) ns	Married	287 (8,434)	3.40% (3.02%-3.79%) ***	9 (3-20) ns
	Uninsured	60 (1,310)	4.58% (3.45%-5.71%)	5 (1-11)	Unmarried	516 (10,928)	4.72% (4.32%-5.12%)	6 (2-15)
	Unk	19 (593)	3.20% (1.79%-4.62%)	19 (3-39)	Unk	41 (1,296)	3.16% (2.21%-4.12%)	8 (3-19)
Other GYN	Insured	428 (9,884)	4.33% (3.93%-4.73%) *	5 (1-23) ns	Married	184 (4,337)	4.24% (3.64%-4.84%) ns	7 (1-35) *
	Uninsured	20 (290)	6.90% (3.98%-9.81%)	2 (0-11)	Unmarried	251 (5,436)	4.62% (4.06%-5.18%)	4 (1-16)
	Unk	9 (340)	2.65% (0.94%-4.35%)	1 (0.5-7)	Unk	22 (741)	2.97% (1.75%-4.19%)	2 (0-14)
Bone tumor	Insured	582 (4,793)	12.14% (11.22%-13.07%) *	19 (8-NA) ***	Married	167 (1,775)	9.41% (8.05%-10.77%) ***	9 (3-25) ***

	Uninsured	23 (178)	12.92% (7.99%-17.85%)	10 (3-NA)	Unmarried	423 (3,081)	13.73% (12.51%-14.94%)	25 (10-NA)
	Unk	4 (108)	NA	4 (2-11)	Unk	19 (223)	8.52% (4.86%-12.18%)	16 (6-38)
STS	Insured	2,049 (22,964)	8.92% (8.55%-9.29%) ***	8 (2-23) ns	Married	1,002 (12,283)	8.16% (7.67%-8.64%) ***	9 (2-24) ns
	Uninsured	103 (933)	11.04% (9.03%-13.05%)	9 (2-22)	Unmarried	1,095 (10,738)	10.20% (9.63%-10.77%)	8 (2-23)
	Unk	29 (1,196)	2.42% (1.55%-3.30%)	4 (1-28)	Unk	84 (2,072)	4.05% (3.20%-4.90%)	5 (1-16)
Skin melanoma	Insured	985 (80,810)	1.22% (1.14%-1.29%) ***	6 (2-20) ns	Married	541 (52,158)	1.04% (0.95%-1.12%) ***	7 (2-23) ***
	Uninsured	44 (1,676)	2.63% (1.86%-3.39%)	4 (1-12)	Unmarried	472 (24,950)	1.89% (1.72%-2.06%)	5 (2-16)
	Unk	36 (24,801)	0.15% (0.10%-0.19%)	7 (1-25)	Unk	52 (30,179)	0.17% (0.13%-0.22%)	10 (4-31)
Non-skin melanoma	Insured	95 (2,901)	3.27% (2.63%-3.92%) *	6 (3-14) ns	Married	54 (1,662)	3.25% (2.40%-4.10%) ns	6 (3-10) ns
	Uninsured	8 (89)	8.99% (3.05%-14.93%)	5.5 (4.5-6.5)	Unmarried	46 (1,150)	4.00% (2.87%-5.13%)	6 (3-18)
	Unk	3 (94)	NA	8 (6-16)	Unk	6 (272)	2.21% (0.46%-3.95%)	5.5 (3-6)
Skin cancer	Insured	32 (4,540)	0.70% (0.46%-0.95%) ns	9 (5-17) ns	Married	17 (2,664)	0.64% (0.34%-0.94%) ns	9 (5-11) ns
	Uninsured	1 (65)	NA	NA (NA-NA)	Unmarried	17 (1,648)	1.03% (0.54%-1.52%)	17 (7-34)
	Unk	4 (740)	NA	35 (7-NA)	Unk	3 (1,033)	NA	14 (8-20)
Embryonal tumors	Insured	325 (4,096)	7.93% (7.11%-8.76%) ns	NA (20-NA) ns	Married	12 (357)	3.36% (1.49%-5.23%) ***	6 (3.5-16) ***
	Uninsured	3 (88)	NA	12 (2-NA)	Unmarried	322 (3,876)	8.31% (7.44%-9.18%)	NA (26-NA)
	Unk	6 (97)	6.19% (1.39%-10.98%)	NA (NA-NA)	Unk	0 (48)	NA	NA
All other	Insured	2,694 (30,805)	8.75% (8.43%-9.06%) ***	5 (1-20) *	Married	1,320 (16,211)	8.14% (7.72%-8.56%) ***	6 (1-23) ***
	Uninsured	133 (1,202)	11.06% (9.29%-12.84%)	2 (0-13)	Unmarried	1,443 (15,004)	9.62% (9.15%-10.09%)	4 (1-18)
	Unk	76 (1,061)	7.16% (5.61%-8.71%)	2 (0-14)	Unk	140 (1,853)	7.56% (6.35%-8.76%)	5 (1-14)

[a] Prevalence was only calculated in category with more than 5 sLM cases.

[b] Survival data (in months) are shown as the median survival and interquartile range in cases with synchronous lung metastasis at diagnosis.

[c] Liver cancer here includes hepatocellular carcinoma and intrahepatic cholangiocarcinoma.

Abbreviations: sLM: synchronous lung metastasis; Unk: unknown; GI: gastrointestinal cancer; GU: genitourinary cancer; GYN: gynecologic cancer; STS: soft-tissue sarcoma; NA: non-applicable; ns: non-significant; *: p<0.05; ***: p<0.001% for intragroup survival comparison by Chi-square tests or log-rank tests.
